# T2R bitter taste receptors regulate apoptosis and may be associated with survival in head and neck squamous cell carcinoma

**DOI:** 10.1002/1878-0261.13131

**Published:** 2021-12-14

**Authors:** Ryan M. Carey, Derek B. McMahon, Zoey A. Miller, TaeBeom Kim, Karthik Rajasekaran, Indiwari Gopallawa, Jason G. Newman, Devraj Basu, Kevin T. Nead, Elizabeth A. White, Robert J. Lee

**Affiliations:** ^1^ Department of Otorhinolaryngology University of Pennsylvania Perelman School of Medicine Philadelphia PA USA; ^2^ Department of Epidemiology Division of Cancer Prevention and Population Sciences The University of Texas MD Anderson Cancer Center Houston TX USA; ^3^ Department of Radiation Oncology The University of Texas MD Anderson Cancer Center Houston TX USA; ^4^ Department of Physiology University of Pennsylvania Perelman School of Medicine Philadelphia PA USA

**Keywords:** apoptosis, calcium, cell signaling, genetics, head and neck squamous cell carcinoma, taste receptors

## Abstract

Better management of head and neck squamous cell carcinomas (HNSCCs) requires a clearer understanding of tumor biology and disease risk. Bitter taste receptors (T2Rs) have been studied in several cancers, including thyroid, salivary, and GI, but their role in HNSCC has not been explored. We found that HNSCC patient samples and cell lines expressed functional T2Rs on both the cell and nuclear membranes. Bitter compounds, including bacterial metabolites, activated T2R‐mediated nuclear Ca^2+^ responses leading to mitochondrial depolarization, caspase activation, and ultimately apoptosis. Buffering nuclear Ca^2+^ elevation blocked caspase activation. Furthermore, increased expression of T2Rs in HNSCCs from The Cancer Genome Atlas is associated with improved overall survival. This work suggests that T2Rs are potential biomarkers to predict outcomes and guide treatment selection, may be leveraged as therapeutic targets to stimulate tumor apoptosis, and may mediate tumor‐microbiome crosstalk in HNSCC.

## Graphical abstract

Head and neck squamous cell carcinoma (HNSCC) cells express bitter taste receptors (T2Rs) on the cell and nuclear membranes. T2Rs are activated by various bitter compounds including secreted bacterial products, leading to nuclear calcium (Ca^2+^) responses, caspase activation, mitochondrial depolarization, and apoptosis. HNSCC tumors that express higher levels of T2Rs are associated with improved overall survival. Created with BioRender.

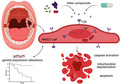

**Abbreviations**
3‐oxo‐C12HSL: N‐3‐oxo‐dodecanoyl‐L‐homoserine lactone
Ca^2+^
: calcium
Ca^2+^_i_
: intracellular calcium
Ca^2+^_nuc_
: nuclear calcium
eCFP: enhanced cyan fluorescent protein
ER: endoplasmic reticulum
FRET: Förster resonance energy transfer
GFP: G protein‐coupled receptors
HNSCC: head and neck squamous cell carcinoma
HPV: human papillomavirus
HPV−: human papillomavirus negative
HPV+: human papillomavirus positive
IP_3_
: inositol trisphosphate
IP_3_R: inositol trisphosphate receptor
NO: nitric oxide
PLC: phospholipase C
PQS: *Pseudomonas* quinolone signal
PTC: phenylthiocarbamide
qPCR: quantitative reverse transcription PCR
R‐GECO‐nls: nuclear‐localized genetically encoded calcium biosensor
T2R: bitter taste receptor protein
*TAS2R*
: bitter taste receptor gene
TCGA: The Cancer Genome Atlas
TMRE: tetramethylrhodamine ethyl ester
ΔΨm: mitochondrial membrane potential

## 1 Introduction

Head and neck squamous cell carcinoma (HNSCC) is the 6th most common cancer worldwide, with an expected 30% incidence increase by 2030 [1]. HNSCC often presents with locally advanced disease [2], and approximately half of patients die ≤ 5 years after diagnosis [3]. Treatment is based on clinical and pathologic risk factors, typically consisting of a combination of surgery, radiation, chemotherapy, and immunotherapy in select cases [4, 5]. Because treatment side effects impact quality of life, it is important to tailor the aggressiveness of therapy to disease risk [6].

HNSCCs associated with human papillomavirus (HPV) show improved overall survival and recurrence‐free survival compared with HPV‐negative tumors. Thus, HPV status is an important tool for risk stratification and treatment selection [7, 8]. There is need for additional methods or biomarkers for stratifying disease risk. There is also a need for alternative therapies that maximize survival while minimizing morbidity in HNSCC. The ideal cancer therapy may be one that exploits cellular machinery to prevent immune system evasion or impact tumor microenvironment.

The current study suggests that bitter taste receptors (T2Rs) may be important for risk stratification and/or as novel therapeutic targets in HNSCC. Initially identified on the tongue, T2Rs are expressed in other tissues, including the gastrointestinal [9] and airway epithelia [10, 11]. There are 25 human T2R isoforms [10] encoded by bitter taste receptor gene (*TAS2R*) genes [12]. T2Rs are G protein‐coupled receptors (GPCRs) that signal through Gα‐mediated cAMP decrease and Gβγ activation of phospholipase C (PLC) and calcium (Ca^2+^) release [10]. T2Rs serve a diverse array of chemosensory functions, including in innate immunity [10, 13]. For example, T2Rs in nasal cells bind bacterial products to activate Ca^2+^‐driven nitric oxide (NO) production to increase cilia beating and kill bacteria [10, 11].

T2Rs have also been explored in some cancers [14, 15, 16, 17, 18, 19, 20, 21, 22, 23], including thyroid [17], salivary [18], and gastrointestinal cancers [14, 15, 16, 19, 22, 23]. Polymorphisms in the *TA2R38* gene encoding the T2R38 receptor are associated with elevated cancer risk [15, 16, 23, 24]. Individuals with specific *TAS2R3* and *4* haplotypes may have lower risk of papillary thyroid cancer [17]. Recent work demonstrated elevated expression of many GPCRs in solid tumors compared with normal tissue [25]. While there is prior work on T2Rs in cancer, an association between T2Rs and HNSCC and the potential T2R signaling pathways within cancer cells are unknown.

We hypothesized that HNSCCs may differentially express *TAS2Rs* compared with normal tissue and some T2Rs are functional and regulate cellular processes. We characterized *TAS2R* expression in HNSCC patient samples and cell lines. We demonstrate that some T2Rs have intracellular or intranuclear localization and can be activated to cause mitochondrial depolarization and apoptosis in HNSCC cells. We subsequently evaluated HNSCC patients for association of *TAS2R* expression levels with survival outcomes using The Cancer Genome Atlas (TCGA).

## 2 Materials and methods

Unless noted, all reagents and protocols were used as previously described [11, 26, 27, 28, 29]. All reagents and catalogue numbers are in Table S1.

### 2.1 Cell culture

SCC4, SCC15, SCC90, and SCC152 cells were from ATCC (Manassas, VA USA). UMSCC47 (SCC47) was from Sigma‐Aldrich (St. Louis, MO USA). OCTT2 cell line was derived from a surgical specimen of an oral SCC tumor [30]. VU147T was from Dr. Hans Joenje, VU Medical Center, the Netherlands. All cancer cell lines were grown in submersion in high glucose Dulbecco's modified Eagle medium (Gibco; Gaithersburg, MD, USA) plus 10% FBS, penicillin/streptomycin mix (Gibco), and nonessential amino acids (Gibco). Primary gingival keratinocytes were from ATCC (Manassas, VA, USA) and used within 2 passages using dermal cell basal keratinocyte medium (ATCC).

### 2.2 Patient samples

This study was approved by the University of Pennsylvania Institutional Review Board (protocol #417200). All subjects provided written informed consent for study participation. SCC specimens were obtained from patients undergoing diagnostic biopsies of HNSCC tumors as part of routine clinical care. Tissue acquisition was done in accordance with the University of Pennsylvania guidelines for the use of residual clinical material and in accordance with the U.S. Department of Health and Human Services code of federal regulation Title 45 CFR 46.116 and the Declaration of Helsinki. Tumor and contralateral normal tissue was obtained. Specimens were divided for pathologic evaluation, and expression analysis samples were collected in TRIzol (Thermo Fisher Scientific).

All tumor specimens had final pathology consistent with SCC. HPV positivity was determined after p16 testing, based on immunohistochemistry that demonstrated ≥ 70% of tumor nuclear and cytoplasmic staining [31]. Testing was performed on cancers of the oropharynx, but not for cancers of other HNSCC sites per the University of Pennsylvania guidelines.

### 2.3 Quantitative reverse transcription PCR

Patient samples and subconfluent cultures were resuspended in TRIzol (Thermo Fisher Scientific). RNA was isolated and purified (Direct‐zol RNA kit; Zymo Research), reverse transcribed via High‐Capacity cDNA Reverse Transcription Kit (Thermo Fisher Scientific), and quantified using TaqMan qPCR probes for the 25 *TAS2R* genes and UBC (QuantStudio 5; Thermo Fisher Scientific). The expression of each *TAS2R* gene was normalized to housekeeping UBC gene, which has been shown to be a stable housekeeping gene in many cancer cell lines [32, 33].

### 2.4 Live cell imaging

Cells were loaded with 5 μm of Fluo‐4‐AM or Fluo‐8‐AM for 45 min at room temperature in the dark, then imaged using an Olympus IX‐83 microscope (20x 0.75 NA PlanApo objective), FITC filters, Orca Flash 4.0 sCMOS camera (Hamamatsu, Tokyo, Japan), MetaFluor (Molecular Devices, Sunnyvale, CA USA), and XCite 120 LED Boost (Excelitas Technologies). For nuclear Ca^2+^, R‐GECO‐nls [34] was transfected using Lipofectamine 3000 (Thermo Fisher Scientific) 24–48 h prior to imaging. Live cell images were taken as above with standard TRITC filters.

For Flip‐GFP, cells were transfected and imaging was carried out on an Olympus IX‐83 microscope as above with 10x (0.4 NA) or 4x (0.16 NA) objective and FITC and TRITC filter sets. For CFP‐DEVD‐mVenus, cells were imaged at 40x (0.75NA objective) with CFP/YFP filters (Chroma 89002‐ET‐ECFP/EYFP) in excitation and emission filter wheels (Sutter Lambda LS). Images were acquired at 37 °C using a stage‐top incubator (Tokai Hit, Tokyo, Japan).

### 2.5 Immunofluorescence

Cultures were fixed in 4% paraformaldehyde for 20 min at room temperature, followed by blocking and permeabilization in DPBS containing 5% normal donkey serum, 1% BSA, 0.2% saponin, and 0.1% Triton X‐100 for 45 min. Cultures were with T2R or tubulin antibodies (1 : 100) at 4 °C overnight. Several T2R antibodies (T2R14, T2R4) were validated previously [11, 13, 35]. Cultures were then incubated with AlexaFluor‐labeled donkey secondary antibodies (1 : 1000) at 4 °C for 1 h and then mounted with Fluoroshield with DAPI (Abcam, Cambridge, UK). Images were obtained using an Olympus IX‐83 microscope (60x 1.4 NA oil; metamorph software, San Jose, CA, USA).

### 2.6 Mitochondrial membrane potential and apoptosis measurements

JC‐1 dye was added to subconfluent cells on 24‐well glass‐bottom plates (CellVis, Mountain View, CA, USA) 10 min prior to measurements (ex.488/em.535 and em.590). CellEvent Caspase 3/7 was added directly prior to measurements (ex.495/em.540) per manufacturer’s specifications. XTT was added directly prior to measurements at 475 and 660 nm. All data for JC‐1, CellEvent Caspase 3/7, and XTT assays were obtained Tecan (Männedorf, Switzerland) Spark 10M. TMRE, Red Dot 2, and Hoechst were added per manufacturer’s specifications. Data were obtained using an Olympus IX‐83 microscope (20x 0.8 NA objective; MetaFluor, MetaMorph, San Jose, CA, USA) with DAPI, TRITC, and Cy5 filters.

### 2.7 The Cancer Genome Atlas analysis

Data were obtained from the PanCancer Atlas and Firehose Legacy datasets of TCGA from cBio Cancer Genomics Portal (cbioportal.org) [36, 37]. The PanCancer Atlas dataset was used for comparisons of *TAS2R* mRNA expression in tumor and adjacent normal tissue. These data were derived from the relative expression of a gene in a tumor sample compared with the expression distribution of all adjacent normal tissue samples in the cohort. The data were provided as an expression *z*‐score which is the number of standard deviations away from the mean of expression in the normal tissue reference population. The tumor‐normal expression analysis was conducted by selecting tumors with ICD codes corresponding to the anatomic sites of oropharynx (C09.9, C01.9, C10.9) and oral cavity (C02.9, C04.9, C06.0, C03.9, C00.9, C14.8). Tumors were further stratified by HPV status based on expression of viral E6/E7 as previously described [38]. The database was queried for all detectable *TAS2R* genes.

The larger Firehose Legacy dataset was used for all other TCGA analyses of *TAS2R*s. We included all HNSCC samples with mutation and copy number alteration data available. Expression comparisons were based on each queried gene compared with that gene's expression in a reference population consisting of all samples that are diploid for the gene [37].

### 2.8 Data analysis and statistics

*t*‐Tests (two comparisons only) and one‐way ANOVA (>2 comparisons) were calculated using graphpad prism (San Diego, CA, USA) with appropriate posttests, as indicated. Additional data analysis was performed using the cbio portal software, Microsoft Excel, or R version 4.0.3 (2020‐10‐10). All figures used the following annotations: *P* < 0.05 (*), *P* < 0.01 (**), *P* < 0.001 (***), and no statistical significance (ns). All data points represent the mean ± SEM.

## 3 Results

### 3.1 T2Rs are differentially expressed and localized in HNSCC

**Fig. 1:**
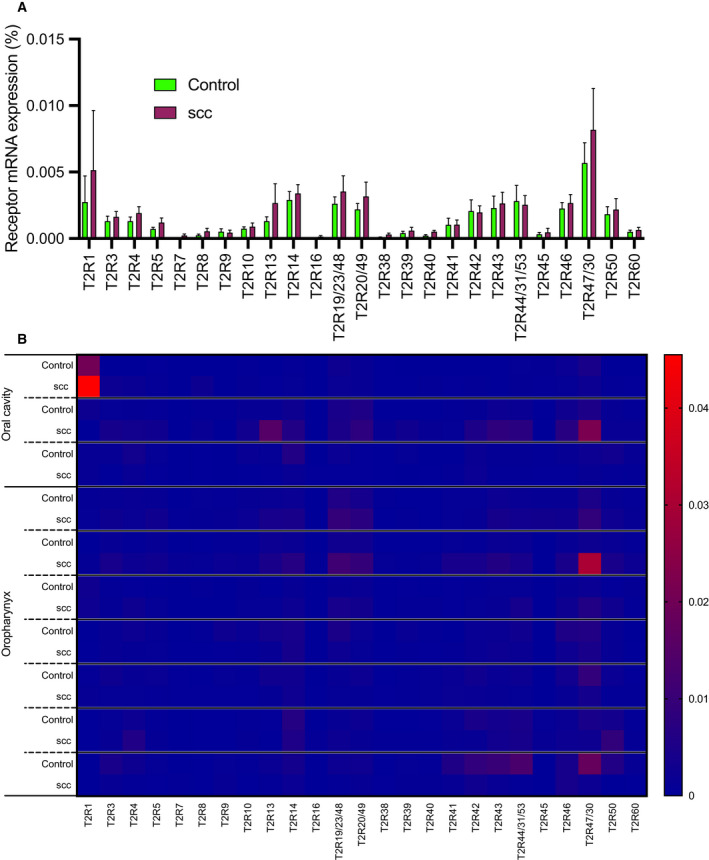
There is variable mRNA expression of bitter (T2R) taste receptor genes in HNSCC. (A) Quantitative PCR (qPCR) expression analysis of T2R transcripts from biopsy specimens of HNSCC (‘SCC’) compared with contralateral normal tissue (‘control’) from the same patient (mean ± SEM; 10 patients). Expression of each *TAS2R* mRNA was normalized to ubiquitin C (UBC) housekeeping gene. No significant differences in individual *TAS2R* gene expression between cancer and control samples by two‐way ANOVA with Bonferroni posttest. (B) Heatmap representation of the same T2R transcript data, grouped by individual patients.

We examined expression of 25 *TAS2Rs* in HNSCC tissue from patients undergoing diagnostic biopsies during routine clinical care. Tissue was obtained from the tumor site and corresponding contralateral normal site from 10 patients (Table S2). Quantitative reverse transcription PCR (qPCR) demonstrated variable expression of *TAS2R*s (Fig. 1A,B). In aggregate, there were no significant differences in expression of individual *TAS2R*s between control and cancer samples (Fig. 1A). However, comparison at the individual patient level demonstrated that some individuals had increased *TAS2R* expression in the tumor specimens while others had decreased expression compared with matched control tissue (Fig. 1B). This small sample size suggests that T2Rs are expressed in HNSCC tissue, but was not robust enough to determine differences in normal vs cancer tissue, including potential associations with clinical outcomes.

*TAS2R* expression was measured via qPCR in HNSCC cell lines SCC4, SCC15, SCC47, SCC90, SCC152, OCTT2, and VU147T (Fig. S1). Like patient samples, cells had variable *TAS2R* expression, but specific *TAS2R*s were consistently high, including *TAS2R4*, *TAS2R14*, *TAS2R19* (also known as *TAS2R23* or *TAS2R48*), *TAS2R20* (also known as *TAS2R49*), and *TAS2R30* (formerly known as *TAS2R47*), *TAS2R43*, and *TAS2R45*. Similarities in *TAS2R* expression between cell lines and tumor specimens suggest these cells may be useful for studying T2R signaling in HNSCC. We also confirmed expression of T2R4, T2R8, T2R10, T2R30, and T2R39 in HNSCC cells by western blot (Fig. S2).

**Fig. 2:**
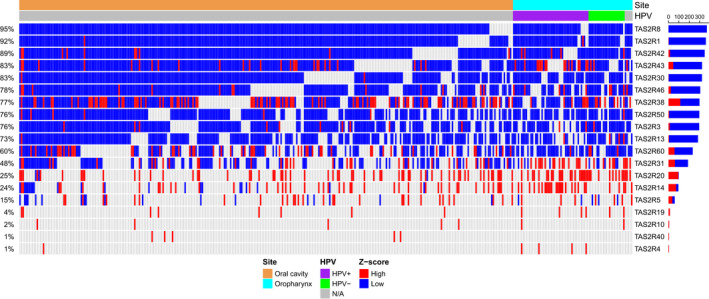
Bitter taste receptor (*TAS2R*) expression in HNSCC is altered compared with normal tissue. *TAS2R* expression was compared for tumor and adjacent normal tissue in 390 cases of HNSCC of the oral cavity and oropharynx using TCGA [34, 35]. *TAS2R* mRNA expression *z*‐scores relative to adjacent normal tissue are shown in the heatmap with *z*‐scores ≥ 2 considered high (red) and *z*‐score ≤ −2 considered low expression (blue). *TAS2R* genes were removed from the plot if they did not have any high or low *z*‐scores across all patients. Data are stratified by oral cavity and oropharynx anatomic sites and by HPV status (HPV+, HPV−, and N/A).

To compare our clinical samples to a larger dataset, we performed an mRNA expression analysis of TCGA for tumor and adjacent normal tissue from 390 patients with HNSCC of the oral cavity and oropharynx (Fig. 2). The *TAS2R* expression was depicted as *z*‐scores calculated relative to normal tissue which indicates how many standard deviations away expression of the tumor tissue lie from the expression of the normal tissue. We find that *TAS2R* expression *z*‐scores tended to be low for several isoforms including *TAS2R8*, *TAS2R1*, and *TAS2R42* which were low in ˜ 90% of patients. In contrast, *z*‐scores for other *TAS2Rs* such as *TAS2R14* and *TAS2R20* were high in ∼ 20%–25% of patients. *TAS2R4* and *TAS2R19* expression *z*‐scores were high for a smaller proportion of patients at ∼ 1% and ∼ 4%, respectively. There were no obvious trends in expression based on anatomic site or HPV status.

**Fig. 3:**
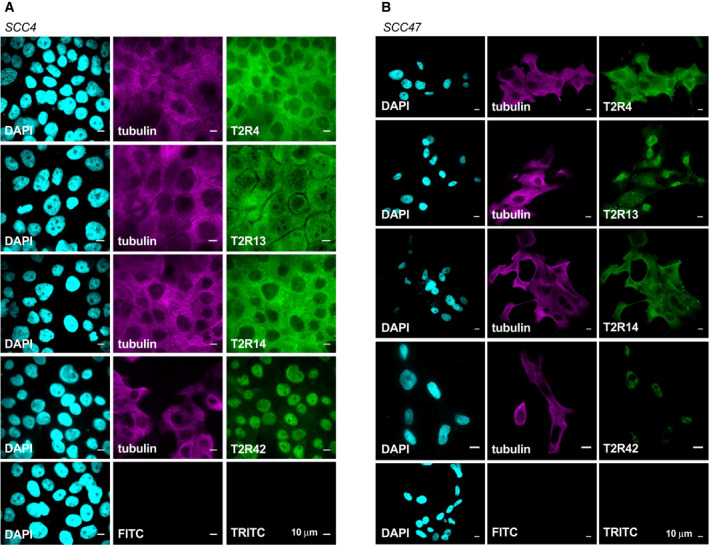
A subset of T2Rs localize to the nucleus of two HNSCC cell lines. Fixed cultures of HNSCC cell lines (A) SCC4 and (B) SCC47 stained with antibodies targeting endogenous proteins demonstrate nuclear localization of T2R42. T2R13 appears to localize strongly to the nucleus in SCC47 but not in SCC4. T2Rs 4 and 14 are expressed in both cell lines and appear to localize to the plasma membrane. For all images, 1 representative image from 3 experiments was shown. Scale bar represents 10 µm. Each antibody was compared to secondary only control at the same microscope settings.

Next, we used confocal immunofluorescence microscopy to visualize T2R localization. Fixed SCC47 and SCC4 cells were stained with antibodies targeting endogenous tubulin and T2Rs (Figs 3 and S3). Interestingly, T2R42 localized to the nucleus in both cell lines and T2R13 was nuclear‐localized in SCC47 but not SCC4. Other T2Rs (including 4, 8, 10, 14, 30/47, and 46) appeared to localize to intracellular membranes, potentially including the endoplasmic reticulum (ER). T2R expression on the nuclear membrane is novel, and fits with our studies of airway cells, where squamous dedifferentiation promotes intracellular and even nuclear localization (preprint [39]).

### 3.2 T2R‐activated nuclear Ca^2+^ responses in HNSCC

**Fig. 4:**
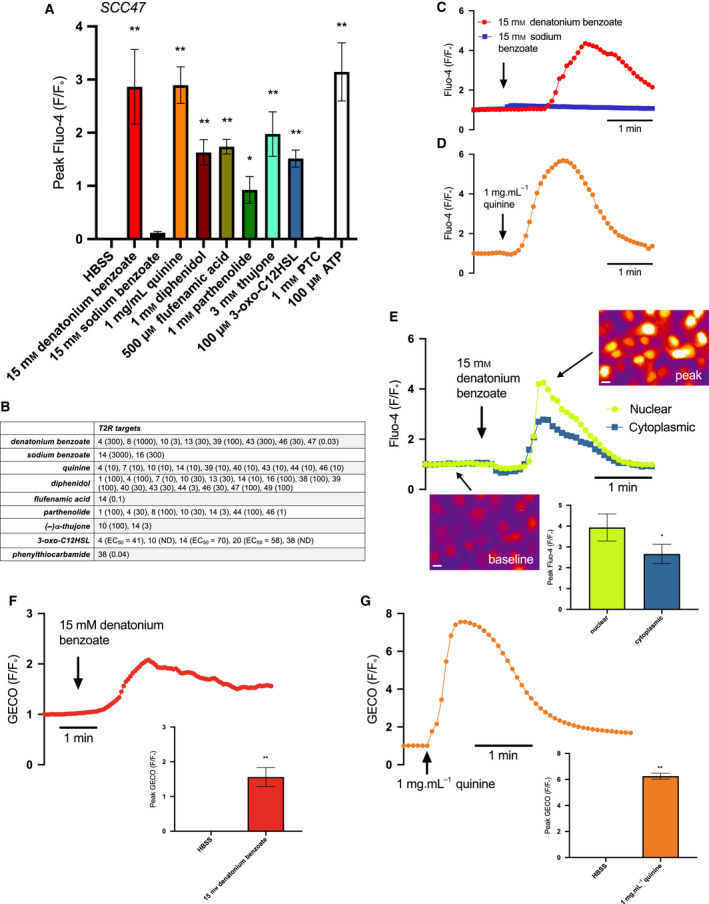
Bitter (T2R) agonists activate Ca^2+^_i_ responses in cultured HNSCC. HNSCC cell line SCC47 loaded with Ca^2+^ binding dye, Fluo‐4, was stimulated with T2R agonists, and Ca^2+^ was measured over time. (A) Peak Fluo‐4 F/F_o_ was quantified after stimulation with Hank's Balanced Salt Solution (HBSS), denatonium benzoate, sodium benzoate, quinine, diphenidol, flufenamic acid, parthenolide, thujone, 3‐oxo‐C12HSL, phenylthiocarbamide (PTC), and purinergic receptor agonist adenosine triphosphate (ATP) (mean ± SEM; 3 experiments using separate cultures). Significance by 1‐way ANOVA with Bonferroni posttest comparing HBSS to each agonist. (B) Table of T2R targets for agonists used. Effective concentration (EC, in µm) or half‐maximal effective concentration (EC_50_, when indicated) shown in parentheses, taken from [40, 50]. Compounds with ‘ND’ denote EC not determined. (C, D) Representative traces of Fluo‐4 F/F_o_ over time after stimulation with denatonium benzoate (C) and sodium benzoate (separate traces superimposed) and quinine (D). Traces are from *n* = 1 culture each but are representative of results obtained from 3 independent cultures (mean ± SEM shown in *A*). (E) Fluo‐4 fluorescence in response to denatonium benzoate‐induced Ca^2+^ release appears to localize more strongly to the nucleus than the cytoplasm as represented by the traces and images; peak Fluo‐4 F/F_o_ for nuclear Ca^2+^ release is significantly greater than cytoplasmic Ca^2+^ release (mean ± SEM; 3 separate cultures). Significance by unpaired *t*‐test. Scale bar is 10 µm. (F, G) Nuclear‐localized R‐GECO‐nls was used to measure nuclear Ca^2+^ after stimulation with (F) denatonium benzoate and (G) quinine. Both agonists stimulate nuclear Ca^2+^ as demonstrated by the traces over time and peak GECO F/F_o_ compared to HBSS (mean ± SEM; 3 separate cultures). Significance by unpaired *t*‐test. *P < 0.05; **P < 0.01.

To determine whether T2Rs in HNSCC cells are functional, we examined agonist‐induced intracellular calcium (Ca^2+^_i_) changes in SCC47, SCC4, SCC15, SCC152, SCC90, OCT22, and VU147T loaded with Ca^2+^ indicator Fluo‐4 (Fig. 4A). We tested bitter compounds targeting multiple T2Rs (Fig. 4B). SCC4 and SCC47 exhibited Ca^2+^_i_ elevations in response to bitter agonists denatonium benzoate, quinine, diphenidol, flufenamic acid, parthenolide, thujone, and the *P. aeruginosa* quorum‐sensing molecule N‐3‐oxo‐dodecanoyl‐L‐homoserine lactone (3‐oxo‐C12HSL, 100 μm; Figs 4A–D and S4). Similarly, SCC15, SCC152, SCC90, OCT22, and VU147T exhibited Ca^2+^_i_ responses to a smaller subset of screened bitter agonists (Fig. S5). None of the cells responded to T2R38 agonist phenylthiocarbamide (PTC), consistent with *TAS2R38* expression at very low levels.

In heterologous expression studies, denatonium benzoate activates ˜ 8 T2Rs (Fig. 4B) while sodium benzoate activates only T2R14 and T2R16 very weakly (mm effective concentrations) [40] (Fig. 4B). Therefore, we used sodium benzoate as a pH and osmolarity control. Denatonium benzoate activated Ca^2+^_i_ responses while equimolar sodium benzoate did not (Fig. 4A,C), suggesting that the observed Ca^2+^_i_ is likely due to activation of specific T2Rs via the denatonium moiety.

Fitting with GPCR activation, denatonium benzoate, and thujone Ca^2+^_i_ responses in SCC4 and SCC47 cells were inhibited by PLC inhibitor U73122 (Fig. S6A–C). Ca^2+^_i_ responses to denatonium benzoate, thujone, and *P. aeruginosa* 3‐oxo‐C12HSL were also blocked by heterotrimeric G protein inhibitor YM254890 [41] (Fig. S6D‐G). Consistent with GPCR‐induced PLC activation, Ca^2+^_i_ responses to denatonium benzoate and quinine were also blocked with inositol trisphosphate (IP_3_) receptor (IP_3_R) inhibitor xestospongin C (Fig. S6H–J). Fluo‐4 responses to denatonium benzoate were significantly reduced in SCC90 cells stably transfected with shRNA plasmids directed against denatonium‐responsive T2R4 (Fig. S7), suggesting T2R4 is important for the denatonium‐induced Ca^2+^_i_ response.

Fluo‐4 responses to denatonium benzoate appeared primarily nuclear (shown for SCC47 in Fig. 4E). This is similar to our studies of airway squamous cells showing T2Rs primarily regulate nuclear calcium (Ca^2+^_nuc_; preprint [39]). To determine whether bitter agonists elevate Ca^2+^_nuc_, SCC47 cells were transfected with nuclear‐localized genetically encoded calcium biosensor (R‐GECO‐nls [34]). Denatonium benzoate (15 mm) and quinine (1 mg·mL^−1^) triggered Ca^2+^_nuc_ elevation (Fig. 4F,G). Thus, T2Rs in HNSCC cells respond to a wide range of bitter agonists to trigger intracellular and intranuclear Ca^2+^ via G proteins and PLC.

R‐GECO‐nls Ca^2+^_nuc_ responses to denatonium benzoate were reduced in cells cotransfected with *TAS2R4* shRNA but not *TAS2R14* shRNA (Fig. S8A,C). T2R14 does not respond to denatonium benzoate [40]. Conversely, Ca^2+^_nuc_ responses to T2R14‐specific agonist FFA were reduced with cotransfection of *TAS2R14* shRNAs but not *TAS2R4* shRNAs (Fig. S8B,C). Denatonium‐induced Ca^2+^_nuc_ responses were also inhibited by U73122 and YM254980 (Fig. S8D). We noted that Ca^2+^_nuc_ responses were twofold to threefold greater in oral cancer cell lines than in primary oral keratinocytes (Fig. S9).

### 3.3 Nuclear Ca^2+^ leads to mitochondrial dysfunction and apoptosis

**Fig. 5:**
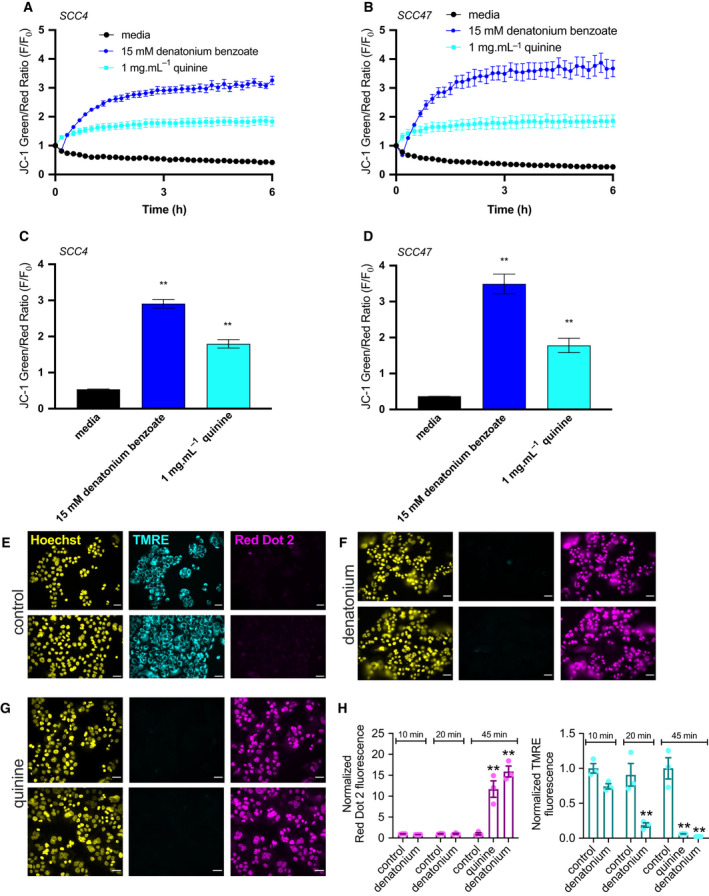
Bitter (T2R) agonists cause mitochondrial depolarization of HNSCC. JC‐1 dye, an indicator of ΔΨ_m_, shows higher green/red signal with depolarized mitochondria. (A–D) SCC cell lines loaded with JC‐1 were stimulated with T2R agonists. Representative traces over 6 h for HNSCC cell lines SCC4 (A) and SCC47 (B) after stimulation with media, denatonium benzoate, and quinine demonstrate mitochondrial depolarization with bitter agonists (mean ± SEM; 3 separate cultures for each cell line). JC‐1 green/red ratio was quantified for SCC4 (C) and SCC47 (D) at 3 h showing significantly higher ratios for denatonium benzoate and quinine compared with media (mean ± SEM; 3 separate cultures for each cell line). Significance by 1‐way ANOVA with Bonferroni posttest comparing media to each agonist. (E–H) SCC4 was treated with media (E), denatonium benzoate (F), and quinine (G) for 45 min then stained with Hoechst (nuclei), TMRE (mitochondria with intact membrane potential), and Red Dot 2 (indicates permeable plasma membrane) dyes. Scale bars in *E–G* are 20 µm. (H) TMRE and Red Dot 2 fluorescence intensities were separately compared between media, denatonium benzoate, and quinine, demonstrating mitochondrial depolarization and plasma membrane permeability after bitter agonist stimulation (mean ± SEM; 3 separate cultures). Significance by 1‐way ANOVA with Dunnett’s posttest comparing media to each agonist. **P < 0.01.

In some cells loaded with Fluo‐8, we visualized an initial Ca^2+^_nuc_ response that was followed by a sustained perinuclear response that appeared mitochondrial (Fig. S10). This led us to investigate how bitter agonists affect mitochondrial function in HNSCC cells. Ca^2+^_nuc_ may feed Ca^2+^ to the mitochondria and regulate metabolism or apoptosis [42]. We loaded SCC4 and 47 cells with ratiometric mitochondrial membrane potential (ΔΨ_m_) dye JC‐1 and exposed them to denatonium benzoate or quinine over 6 h. Both depolarized ΔΨ_m_, evidenced by a shift in the green‐to‐red fluorescence ratio (Fig. 5A–D). We also tested ΔΨ_m_ using tetramethylrhodamine ethyl ester (TMRE), which binds only to mitochondria with intact membrane potential. SCC4 cells were treated with denatonium benzoate and quinine for different lengths of time, stained with TMRE and Red Dot 2, and fluorescence intensities were compared (Fig. 5E–H). Both bitter agonists led to mitochondrial depolarization and subsequent plasma membrane permeability. Like Ca^2+^_nuc_ responses, denatonium‐induced changes in TMRE and JC‐1 fluorescence were inhibited by GPCR Ca^2+^ signaling inhibitors U73122 and YM254890 (Fig. S11A–C).

**Fig. 6:**
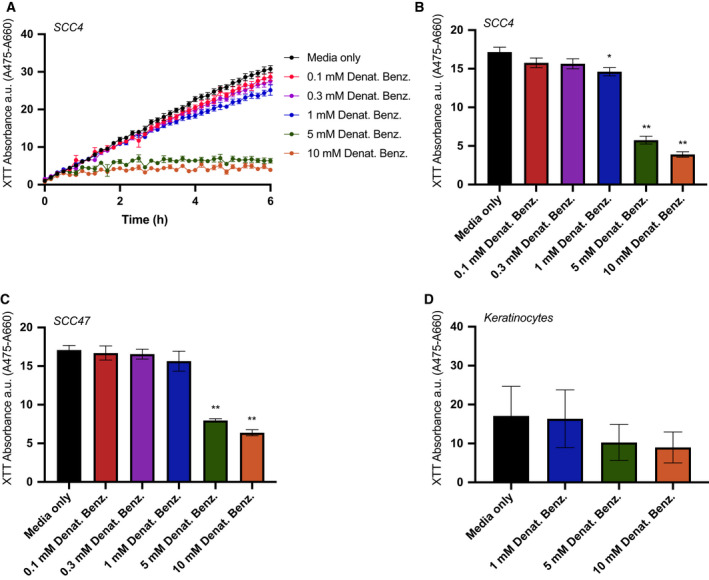
Bitter (T2R) agonists decrease cellular metabolism in HNSCC. 2,3‐bis‐(2‐methoxy‐4‐nitro‐5‐sulfophenyl)‐2H‐tetrazolium‐5‐carboxanilide (XTT) was used to measure cellular NAD(P)H production via a cross‐plasma‐membrane electron transfer. (A) Representative trace over 6 h after stimulation of HNSCC cell line SCC4 with media ± denatonium benzoate at five different concentrations as indicated (mean ± SEM; *n* = 6 independent cultures). (B, C) Bar graphs (mean ± SEM; *n* = 6 independent cultures per cell line) of XTT absorbance quantified at 3 h for (B) SCC4 and (C) SCC47. (D) XTT absorbance quantified at 3 h for primary oral keratinocytes showing smaller (not statistically significant) reduction in NAD(P)H production with denatonium compared with HNSCC cells (*n* = 6 independent cultures from different patients). Significance by 1‐way ANOVA with Dunnett’s posttest comparing media to each concentration of denatonium benzoate. *P < 0.05, **P < 0.01.

Bitter agonists also decreased cellular metabolism, evidenced by a reduction in NAD(P)H production measured by XTT. In the presence of a cofactor, extracellular XTT is reduced from a nonlight‐absorbing to a colored absorbing form by cellular NAD(P)H via cross‐plasma‐membrane electron transfer. We saw blunted XTT changes 3 h in the presence of 1–10 mm denatonium benzoate in SCC4 and 5–10 mm denatonium benzoate in SCC47 cells. Primary oral keratinocytes did not exhibit significant changes in XTT at 5–10 mm denatonium benzoate (Fig. 6), fitting with reduced Ca^2+^_nuc_ responses (Fig. S9).

**Fig. 7:**
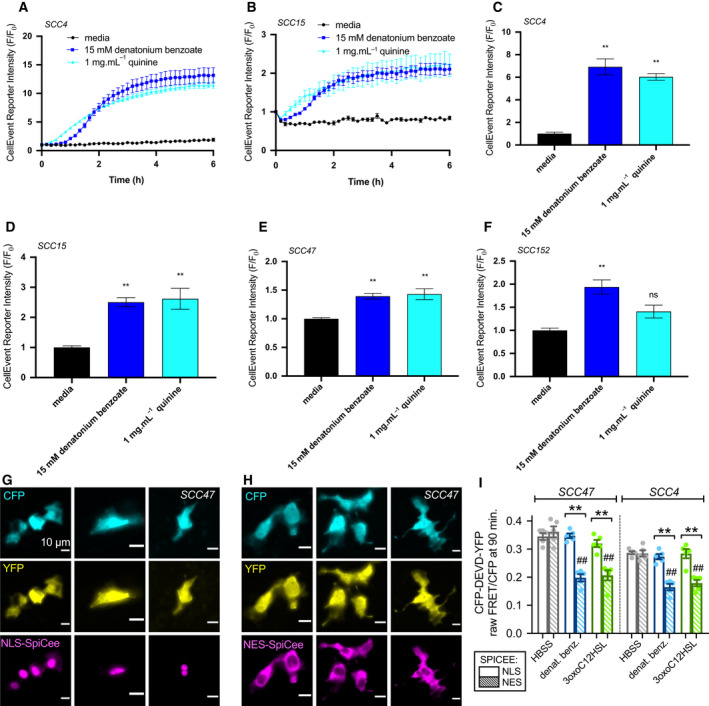
Bitter (T2R) agonists cause activation of apoptosis in HNSCC. (A, B) Cleavage of caspase 3/7 CellEvent reagent signals apoptosis. Representative traces over 6 h after stimulation of HNSCC cell lines SCC4 (A) and SCC15 (B) with media, denatonium benzoate, and quinine demonstrate increased caspase 3/7 cleavage (mean ± SEM; 3 separate cultures for SCC4 and 5 separate cultures for SCC15). (C–F) CellEvent reporter intensity at 6 h after stimulation of SCC4 (C), SCC15 (D), SCC47 (E), and SCC152 (F) with bitter agonists, normalized to media (mean ± SEM; 3 separate cultures for SCC4, SCC47, and SCC152 and 5 separate cultures for SCC15). Significance by 1‐way ANOVA with Bonferroni posttest comparing media to each agonist; **P < 0.01, ns = nonsignificant. (G–H) Wide‐field 40x fluorescence images of mCherry‐labeled NLS‐SpiCee (G) or NES‐SpiCee (H) cotransfected with caspase FRET biosensor in SCC47 cells. Each column shows cells from a separate individual experiment (*n* = 3 for each condition shown), but results representative of results from 5 independent experiments (averaged in I). Scale bars in G and H are 10 µm. (I) FRET ratios were measured in cells after 90‐min incubation with HBSS (gray bars), 10 mm denatonium benzoate (blue bars), or 100 µm 3‐oxo‐C12HSL (green bars). Open bars are NLS‐SpiCee, and crossed bars are NES‐SpiCee. A downward deflection indicates loss of FRET signal signifying caspase cleavage. Each data point is an independent experiment. Graph shows mean ± SEM of 5 independent experiments with each cell line. Each independent experiment imaged 5–9 cells from a single field of view. Significance by one‐way ANOVA with Bonferroni posttest; **P < 0.01 between bracketed bars; ^##^ *P* < 0.01 compared with HBSS.

To test whether mitochondrial impairment led to apoptosis, we treated HNSCC cells with a caspase 3/7‐sensitive dye (CellEvent caspase 3/7 reagent, Thermo). Exposure to denatonium benzoate caused a significant increase in caspase 3/7 activity over 6 h in SCC4, SCC15, SCC47, and SCC152. Quinine led to a significant increase in SCC4, SCC15, and SCC47, but not SCC152 (Fig. 7). Denatonium‐induced caspase 3/7 activity was reduced in the presence of U73122 or YM254890 (Figure S11D,E). Notably, neither denatonium benzoate nor quinine caused significant changes in TMRE fluorescence (measuring mitochondrial potential; Fig. S12A–E) or CellEvent reagent fluorescence (measuring caspase 3/7 activation; Fig. S12F–H) over the same time range that changes were observed with HNSCC cells.

Denatonium benzoate but not sodium benzoate‐induced caspase activation was confirmed in HNSCC cells using an optical assay (Flip‐GFP; [43]) in SCC4, SCC47, SCC90, and SCC152 cells (Figs S13–S14). Changes in Flip‐GFP fluorescence, signaling caspase activation, were blocked by U73122, XeC, or YM254890 (Fig. S14C,D) as well as cotransfection with *TAS2R4* shRNA (Fig. S14E,F).

We further confirmed caspase activation in SCC4 and SCC47 cells using a Förster resonance energy transfer (FRET)‐based biosensor created by linking enhanced cyan fluorescent protein (eCFP) and yellow fluorescent protein variant mVenus with a linker containing a DEVD caspase 3 cleavage site [44]. Caspase cleavage of the protein allows the eCFP and mVenus to diffuse farther apart, reducing FRET (Fig. S15A). We saw FRET decreases signaling caspase activation in response to denatonium benzoate, quinine, thujone, 3‐oxo‐C12HSL, and *Pseudomonas* quinolone signal (PQS) but not sodium benzoate (Fig. S15B–F). In this assay, denatonium benzoate or flufenamic acid‐induced caspase activation was reduced by shRNAs against *TAS2R4* or *TAS2R14*, respectively (Fig. S15G–I). Denatonium benzoate‐induced executioner caspase activation was also confirmed by western blot for cleaved caspases 3 and 7 (Fig. S16).

Loading SCC4 or SCC47 cells with calcium chelator BAPTA blocked caspase activation, measured by Flip‐GFP, in response to denatonium benzoate (Fig. S13G). To determine whether apoptosis was linked to Ca^2+^_nuc_, we utilized a recombinant protein calcium buffer, known as SpiCee [45], fused to either nuclear export or nuclear localization sequences (NES‐SpiCee or NLS‐SpiCee, respectively). NLS‐SpiCee or NES‐SpiCee was cotransfected with the eCFP‐DEVD‐Venus biosensor (Fig. 7G‐H). FRET decreases signaling apoptosis occurred with NES‐SpiCee and bitter agonists denatonium benzoate or bacterial 3‐oxo‐C12HSL (Fig. 7I). However, no apoptosis was observed with NLS‐SpiCee. Chelation of Ca^2+^_nuc_ blocked bitter agonist‐induced apoptosis (Fig. 7I). Bitter agonist‐induced Ca^2+^_nuc_ responses reduce cellular metabolism and proliferation in HNSCC cells.

### 3.4 Increased *TAS2R* expression may be associated with HNSCC survival

**Fig. 8:**
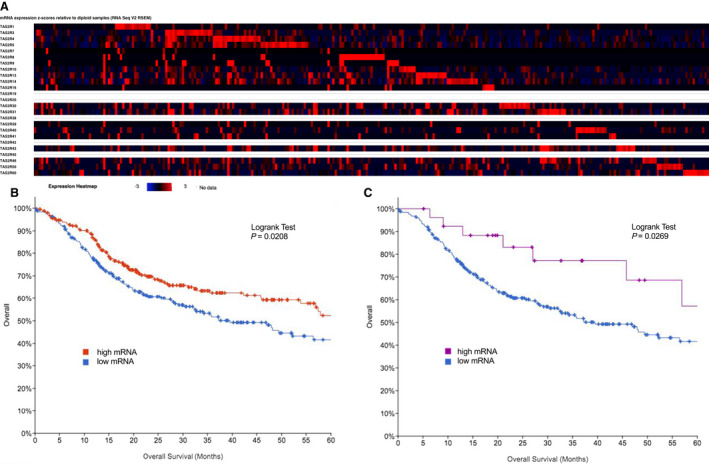
Bitter taste receptor (*TAS2R*) expression alterations in HNSCC are associated with survival. *TAS2R* expression alterations were analyzed for 504 cases of HNSCC using TCGA [36, 37]. (A) Heatmap of *TAS2R* mRNA expression *z*‐scores relative to diploid samples. Increased *TAS2R* expression (red) is more common than decreased expression (blue). (B) 5‐year survival analysis of HNSCC cases demonstrates that increased *TAS2R* expression is associated with improved overall survival (*P* = 0.0208 by log‐rank test). (C) Increased *TAS2R*4 expression, which is activated by the bitter agonists denatonium benzoate, quinine, diphenidol, parthenolide, and 3‐oxo‐C12HSL, is also associated with improved overall survival (*P* = 0.0269 by log‐rank test).

Data above suggest T2Rs activate apoptosis and limit proliferation of HNSCCs *in vitro*, which prompted us to explore possible *in vivo* effects of T2Rs. Specifically, we investigated the impact of increased *TAS2R* expression on survival in HNSCC using TCGA [36, 37]. Analysis included 504 cases of HNSCC diagnosed between 1992 and 2013 with mRNA expression data available. *TAS2R* expression *z*‐scores relative to diploid samples are demonstrated in the heatmap in Fig. 8A. Kaplan–Meier survival analysis of cases with high vs low *TAS2R* expression demonstrated improved overall survival for cases with increased *TAS2R* expression (*P* = 0.0208 by log‐rank test; Fig. 8B). Median survival was 65.77 months for high *TAS2R* expression versus 39.49 months for low mRNA expression. We also separately evaluated *TAS2R4* expression, as this receptor was expressed in the patient samples and cell lines and is activated by denatonium benzoate and quinine. Kaplan–Meier survival analysis comparing high *TAS2R4* expression and low expression demonstrated improved overall survival for cases with increased *TAS2R4* (*P* = 0.0269 by log‐rank test; Fig. 8C). Kaplan–Meier analysis of disease‐free survival for *TAS2R* and *TAS2R4* expression alterations did not show a statistically significant difference (*P* = 0.896 and 0.837 by log‐rank test, respectively; Fig. S17).

Evaluation of genetic changes in addition to expression changes showed that 283 out of 504 cases (56.15%) had some *TAS2R* alteration (Fig. S18A). Multiple alterations were present in 11.51% of cases (Fig. S18B). Kaplan–Meier survival analysis of cases with and without *TAS2R* genetic alterations showed improved survival for patients with genetic alterations which was not statistically significant (*p* = 0.0891 by log‐rank test; Fig. S18C). Similarly, there was improved disease‐free survival for patients with genetic alterations which was not statistically significant (*P* = 0.111 by log‐rank test; Fig. S18D).

TCGA analyses suggest *TAS2R* genetic and expression alterations are prevalent in HNSCC. Increased *TAS2R* expression may be beneficial by limiting cancer cell proliferation via mechanisms outlined above.

## 4 Discussion

While T2Rs have been studied in cancer [14, 15, 16, 17, 18, 19, 20, 21, 22, 23], this is the first description of T2R expression and signaling in HNSCC. Bitter agonists activate T2R‐mediated Ca^2+^_nuc_, which triggers mitochondrial depolarization, caspase activation, and apoptosis. The novel demonstration of a requirement for Ca^2+^_nuc_ in bitter agonist‐induced apoptosis may be important to other cancers. Using TCGA, we report that increased *TAS2R* expression is correlated with HNSCC survival, suggesting potential clinical utility of T2R agonists and supporting future prospective studies in larger patient populations to test if/how T2Rs are diagnostic biomarkers in HNSCC.

The *TAS2Rs* with highest expression in HNSCC (*TAS2R4*, *TAS2R14*, *TAS2R19*, *TAS2R20*, *TAS2R30*, *TAS2R43*, and *TAS2R45*) overlap with *TAS2Rs* expressed at increased levels in breast cancer [20, 21]. However, the concept of T2R expression on the nuclear membrane is highly novel. We (preprint [39]) and others [46] have found nuclear localization of T2Rs in deciliated or inflamed airway tissues. We also saw this in lung cancer cells (preprint [39]). Squamous dedifferentiation likely alters localization of airway T2Rs from cilia to the nucleus, where T2Rs activate Ca^2+^_nuc_ and apoptosis rather than ciliary beating activated in differentiated healthy tissues [11, 35]. HNSCC cells also have at least some nuclear T2Rs. Prior studies have validated importance of nuclear envelope GPCRs in neurons and other cells [47, 48, 49], but the link between intracellular T2Rs, Ca^2+^_nuc_, and apoptosis in cancer cells is novel. Notably, Ca^2+^ can be regulated by GPCRs directly within the nucleus as well as on surrounding ER and/or nuclear envelope membranes ([47, 48, 49]). While more detailed subcellular localization studies are needed, more work is also required to determine potential functional differences of T2R isoforms beyond localization. A detailed study of the localization of all 25 T2R isoforms is needed in future studies.

Interestingly, orphan T2R42 [40, 50] localized to the nucleus in two HNSCC tongue cell lines. Understanding T2R42 function will be difficult with no known agonists, but *TAS2R42* expression in tumor compared with normal tissue was altered in ∼ 89% of oral and oropharyngeal cancers in our TCGA analysis. Understanding whether orphan T2Rs such as T2R42 have endogenous or bacteria‐derived bitter agonists is also critical to interpreting their role in HNSCC cells. Constitutive activity has been described for some mutated T2Rs [51]. One possibility, unsupported but intriguing is that orphan T2Rs may have constitutive activity that plays a role in baseline cell proliferation and/or metabolism.

Further work is needed to determine specific subcellular localizations of each T2R isoform. Combined with knockout studies, this may reveal other novel functions beyond activation of apoptosis in HNSCC cells. Several T2Rs that are activated by denatonium benzoate or quinine (including T2Rs 4, 8, 10, 14, 30/47, and 46) did not localize to the nucleus, but nonetheless appeared intracellular. T2R13, activated by denatonium benzoate [40, 50], was nuclear in SCC47 but not in SCC4. Thus, differences in T2R localization between cancers of the same anatomic site may exist. It is interesting that the denatonium‐induced Ca^2+^ response is strongly dependent on T2R4 despite expression of other denatonium‐responsive T2Rs (e.g., T2R30 and T2R13) in HNSCC cells. It may be that these T2Rs are not linked to Ca^2+^ but are instead linked to other pathways in these cells. Or, it may be that mRNA expression levels do not correlate with protein expression levels. Further work is needed to understand whether all T2R isoforms signal similarly in cancer cells, as much of our knowledge of T2R signaling is based on taste bud cells or heterologous expression systems using chimeric G proteins that force T2Rs to couple to calcium. While useful for screening bitter compounds, these assays cannot reveal differences in endogenous T2R signaling.

Multi‐T2R targeting compounds such as denatonium benzoate or quinine might be useful to activate apoptosis in HNSCCs from a broad range of patients. Many known T2R agonists (e.g., quinine) are cell permeant [52, 53]. Intracellular T2Rs could be targeted using high‐dose topical treatments in accessible anatomic locations like the oral cavity/oropharynx. Many pharmaceuticals with known safety profiles are bitter [54] and could potentially kill or slow the growth of HNSCC, particularly if specific T2R agonists can be used in combination with genetic profiling to determine expression of specific T2Rs in tumor vs normal tissue.

Ca^2+^_nuc_ and cytosolic Ca^2+^ are distinct, but both are activated by ryanodine receptors and IP_3_Rs [55, 56]. Ca^2+^_nuc_ responses to bitter agonists in HNSCC originate partly from the ER, requiring PLC and IP_3_Rs. Nuclear Ca^2+^ regulates transcription factors but was also linked to apoptosis [57, 58, 59]. Bitter agonists activate apoptosis and/or mitochondrial depolarization in other cell types [60, 61, 62, 63], including metastatic breast cancer [64], prostate cancer [65], and acute myeloid leukemia cells [61]. In pancreatic cancer, T2R38 can be activated by 3‐oxo‐C12HSL [19], suggesting a possible link with the microbiome. In contrast, denatonium benzoate had protumor actions in murine submandibular gland cancer cells [18]. We show here that bitter agonists, including bacterial 3‐oxo‐C12HSL and PQS, are antiproliferative and proapoptotic in HNSCC, with a novel mechanistic link between Ca^2+^_nuc_ and apoptosis.

TCGA analysis suggests *TAS2R* expression alterations impact HNSCC or serve as markers of underlying genetic changes that contribute to tumor biology and treatment efficacy. *TAS2R* expression alterations are associated with better survival. We hypothesize that, as T2Rs regulate apoptosis, tumors that overexpress T2Rs may have an improved prognosis due to a more robust internal regulation preventing unchecked proliferation or responding to bitter agonists in the microenvironment, either from the tumor microbiome or intracellular metabolites.

We did not detect an association of *TAS2R* expression and disease‐free survival, suggesting that T2Rs may be associated with longer survival but not necessarily disease control. We also did not appreciate anatomic or HPV‐related differences in *TAS2R* expression, suggesting that expression may vary independently of anatomic site or that an even larger samples size may be needed to detect more subtle differences. Further work is necessary to appreciate the full impact of T2Rs in HNSCC and potential roles in risk stratification and treatment. Furthermore, common genetic polymorphisms in *TAS2Rs* that affect bitter taste perception and dietary preferences [66, 67] may contribute to differences in tumor behavior. Long‐term prospective clinical studies that include multivariate analyses and evaluation of patient and disease factors (e.g., smoking, sex, and tumor stage) are still needed to further clarify the predictive value of T2Rs in HSNCC.

Identification of prognostic markers to guide treatment selection is an active area of HNSCC research to ultimately improve survival while mitigating treatment‐related side effects. Several clinical trials have sought to limit treatment‐related morbidity by de‐intensifying therapy in HPV‐associated oropharyngeal tumors, which are known to have improved prognosis compared with HPV‐negative tumors [68, 69]. If increased *TAS2R* expression portends favorable prognoses, it would be reasonable to perform *TAS2R* expression analysis of HNSCC tumors as part of the treatment selection algorithm, similar to standard practice HPV testing for oropharyngeal SCCs.

Importantly, bacterial ligands such as PQS or 3‐oxo‐C12HSL are hydrophobic and cell permeant [70, 71]. These molecules can reach ≥ 100 µm in localized bacterial environments [72]. Thus, intracellular T2Rs could be activated by these bacterial metabolites. We propose that cancer cell T2Rs may link the host microbiome with HNSCC progression. HNSCCs are known to be associated with unique microbiomes [73, 74, 75, 76, 77], impacting the immune system and contributing to oncogenesis and oncologic outcomes [73, 78, 79]. T2R‐mediated recognition of bacterial ligands and resulting interkingdom signaling may play a role in HNSCC and other cancer pathophysiology.

## 5 Conclusions

Our data demonstrate that HNSCC cells express functional T2Rs on the cell membrane and nucleus which respond to bitter compounds, including bacterial metabolites. Activation of these T2Rs leads to nuclear Ca^2+^ responses, mitochondrial depolarization, caspase activation, and apoptosis. TCGA data suggest that increased expression of *TAS2R*s in HNSCCs is associated with improved overall survival, possibly related to the role of T2Rs in limiting cancer cell proliferation. Future work will be necessary to determine whether this class of receptors can serve as biomarkers for oncologic outcomes or therapeutic targets for selective activation of apoptosis in cancer cells.

## Conflict of interest

The authors declare no conflict of interest.

## Author contributions

RMC, KTN, EAW, and RJL conceptualized and visualized the study; RMC, DBM, ZAM, IG, TBK, and RJL investigated and involved in formal analysis; RMC wrote—original draft; RMC, TBK, EAW, and RJL wrote—review and editing; KR, JGN, and DB curated the data and contributed to resources; RMC and RJL involved in funding acquisition; RJL supervised the study.

## Supporting information


**Table S1.** Catalogue numbers for key biological and chemical reagents used in this study.
**Table S2.** Clinical data for HNSCC patients.
**Fig. S1.** Variable mRNA expression of bitter (T2R) taste receptor genes in HNSCC cell lines.
**Fig. S2.** Confirmation of endogenous T2R protein expression by Western blot.
**Fig. S3.** T2Rs are expressed in HNSCC cell lines.
**Fig. S4.** Bitter (T2R) agonists activate calcium responses in HNSCC cell line.
**Fig. S5.** Bitter (T2R) agonists activate calcium responses in HNSCC.
**Fig. S6.** Pharmacology of the Ca^2+^_i_ response.
**Fig. S7.** Inhibition of denatonium‐induced fluo‐4 Ca^2+^ responses with knockdown of T2R4.
**Fig. S8.** Inhibition of denatonium‐induced or flufenamic acid (FFA)‐induced R‐GECO‐nls Ca^2+^_nuc_ responses with TAS2R4 or TAS2R14 shRNA, respectively.
**Fig. S9.** Reduced Ca^2+^_nuc_ responses in primary oral keratinocytes compared with HNSCC cells.
**Fig. S10.** Nuclear Ca^2+^ responses appeared to propagate to mitochondria in Fluo‐8‐loaded cells.
**Fig. S11.** Inhibition of denatonium‐induced TMRE, JC‐1, and CellEvent changes by GPCR inhibitors.
**Fig. S12.** Lack of effect of T2R stimulation on TMRE, JC‐1, and CellEvent fluorescence in primary keratinocytes.
**Fig. S13.** Confirmation of caspase activation by Flip‐GFP and dependence of denatonium‐induced caspase activation on Ca^2+^ signaling.
**Fig. S14.** Flip‐GFP measurement of caspase activation in response to denatonium benzoate but not sodium benzoate in SCC4, SCC47, SCC90, and SCC152.
**Fig. S15.** Confirmation of bitter agonist‐induced caspase activation by ratiometric caspase biosensor.
**Fig. S16.** Confirmation of denatonium‐induced caspase activation by Western for caspase 3 and 7 cleavage in SCC47 cells.
**Fig. S17.** Disease‐free survival for TAS2R expression alterations in HNSCC.
**Fig. S18.** TAS2R genomic and expression alterations are prevalent in HNSCC.

## Data Availability

All data will be provided under Data Transfer Agreement upon reasonable request to RJL (rjl@pennmedicine.upenn.edu).
