# Supplementary material for: T2R bitter taste receptors regulate apoptosis and may be associated with survival in head and neck squamous cell carcinoma
Source: Mol Oncol. 2021 Dec 14;16(7):1474–92. doi: 10.1002/1878-0261.13131 (PMC8978516; doi:10.1002/1878-0261.13131)
Supplement: Supplementary file 1 — Table S1. Catalogue numbers for key biological and chemical reagents used in this study. Table S2. Clinical data for HNSCC patients. Fig. S1. Variable mRNA expression of bitter (T2R) taste receptor genes in HNSCC cell lines. Fig. S2. Confirmation of endogenous T2R protein expression by Western blot. Fig. S3. T2Rs are expressed in HNSCC cell lines. Fig. S4. Bitter (T2R) agonists activate calcium responses in HNSCC cell line. Fig. S5. Bitter (T2R) agonists activate calcium responses in HNSCC. Fig. S6. Pharmacology of the Ca2+ i response. Fig. S7. Inhibition of denatonium‐induced fluo‐4 Ca2+ responses with knockdown of T2R4. Fig. S8. Inhibition of denatonium‐induced or flufenamic acid (FFA)‐induced R‐GECO‐nls Ca2+ nuc responses with TAS2R4 or TAS2R14 shRNA, respectively. Fig. S9. Reduced Ca2+ nuc responses in primary oral keratinocytes compared with HNSCC cells. Fig. S10. Nuclear Ca2+ responses appeared to propagate to mitochondria in Fluo‐8‐loaded cells. Fig. S11. Inhibition of denatonium‐induced TMRE, JC‐1, and CellEvent changes by GPCR inhibitors. Fig. S12. Lack of effect of T2R stimulation on TMRE, JC‐1, and CellEvent fluorescence in primary keratinocytes. Fig. S13. Confirmation of caspase activation by Flip‐GFP and dependence of denatonium‐induced caspase activation on Ca2+ signaling. Fig. S14. Flip‐GFP measurement of caspase activation in response to denatonium benzoate but not sodium benzoate in SCC4, SCC47, SCC90, and SCC152. Fig. S15. Confirmation of bitter agonist‐induced caspase activation by ratiometric caspase biosensor. Fig. S16. Confirmation of denatonium‐induced caspase activation by Western for caspase 3 and 7 cleavage in SCC47 cells. Fig. S17. Disease‐free survival for TAS2R expression alterations in HNSCC. Fig. S18. TAS2R genomic and expression alterations are prevalent in HNSCC. [file MOL2-16-1474-s001.pdf]

Supplementary Material

Carey, et al. T2R bitter taste receptors regulate apoptosis and may be associated with survival in head and neck squamous cell carcinoma

Supplementary Table S1 Catalogue numbers for key biological and chemical reagents used in this study.

REAGENT or RESOURCE	SOURCE	IDENTIFIER
Antibodies		
anti-T2R8	Abcam	ab75109
anti-T2R13	Abcam	ab172986
anti-T2R4	ThermoFisher Scientific	PA-67752
anti-T2R10	ThermoFisher Scientific	OSR00158W
anti-T2R14	ThermoFisher Scientific	PA5-39710
anti-T2R42	ThermoFisher Scientific	NBP1-83154
anti-T2R30/47	ThermoFisher Scientific	PA5-67773
anti-T2R46	ThermoFisher Scientific	PA5-67772
anti-T2R39	ThermoFisher Scientific	PA5-50670
Alexa Fluor 488-conjugated donkey anti-mouse	ThermoFisher Scientific	A21202
Alexa Fluor 546-conjugated donkey anti-rabbit	ThermoFisher Scientific	A10040
anti-caspase 3 (D3R6Y)	Cell Signaling Technologies	14220
anti-caspase 7 (D2Q3L)	Cell Signaling Technologies	12827
HRP-linked secondary antibody		
anti-α-tubulin	Developmental Studies Hybridoma Bank	12G10
Chemical reagents		
U73122	Cayman Chemical	70740
U73343	Cayman Chemical	17339
YM254890	Cayman Chemical	29735
Xestospongin C	Cayman Chemical	64950
Parthenolide	Cayman Chemical	70080
Diphenhydramine	Cayman Chemical	11158
TMRE (Tetramethylrhodamine ethyl ester)	Cayman Chemical	601283
RedDot 2 Viability Dye	Cayman Chemical	601282
Cell-Based Hoechst Dye	Cayman Chemical	600332
ATP	Millipore Sigma	A9187
(-)-a-Thujone	Millipore Sigma	89231
Flufenamic acid (FFA)	Millipore Sigma	F9005
Denatonium benzoate	Millipore Sigma	D5765
Phenylthiocarbamide (PTC)	Millipore Sigma	P7629
Sodium Benzoate	Millipore Sigma	B3420
Diphenidol	Millipore Sigma	SML2169
Quinine	Millipore Sigma	Q0132

N-3-oxo-dodecanoyl-L-homoserine lactone (3-oxo-C12HSL)	Millipore Sigma	O9139
2-Heptyl-3-hydroxy-4(1H)-quinolone (*Pseudomonas* quinolone signal, PQS)	Millipore Sigma	94398
Saponin	Millipore Sigma	S7900
Bovine Serum Albumin	Millipore Sigma	A2153
TRIzol	ThermoFisher Scientific	15596026
JC-1	ThermoFisher Scientific	T3168
CellEvent^{TM} Caspase-3/7 Green Detection Reagent	ThermoFisher Scientific	C10423
XTT (sodium 3´-[1- (phenylaminocarbonyl)- 3,4-tetrazolium]-bis (4-methoxy6-nitro) benzene sulfonic acid hydrate	ThermoFisher Scientific	X6493
lipofectamine 3000	ThermoFisher Scientific	L3000075
Fluo-4-AM	ThermoFisher Scientific	F14201
Fluo-8-AM	Abcam	ab142773
Normal Donkey Serum	Abcam	ab7475
Critical Commercial Assays		
High-Capacity cDNA Reverse Transcription Kit	ThermoFisher Scientific	4368814
Cells		
VU147T	Hans Joenje, VU Medical Center, Netherlands	N/A
SCC4	ATCC	CRL-1624
SCC15	ATCC	CRL-1623
SCC90	ATCC	CRL-3239
SCC152	ATCC	CRL-3240
UMSCC47 (SCC47)	Millipore Sigma	SCC071
OCTT2	D. Basu, Univ. of Penn [1]	N/A
Primary gingival keratinocytes	ATCC	PCS-200-014
Primers for qPCR		
TaqMan Primers for T2R1	ThermoFisher Scientific	Hs00251930_s1
TaqMan Primers for T2R3	ThermoFisher Scientific	Hs00249942_s1
TaqMan Primers for T2R4	ThermoFisher Scientific	Hs00249946_s1
TaqMan Primers for T2R5	ThermoFisher Scientific	Hs01549633_s1
TaqMan Primers for T2R7	ThermoFisher Scientific	Hs00256778_s1
TaqMan Primers for T2R8	ThermoFisher Scientific	Hs00256766_s1
TaqMan Primers for T2R9	ThermoFisher Scientific	Hs00256757_s1
TaqMan Primers for T2R10	ThermoFisher Scientific	Hs00256794_s1
TaqMan Primers for T2R13	ThermoFisher Scientific	Hs00256781_s1
TaqMan Primers for T2R14	ThermoFisher Scientific	Hs00256800_s1
TaqMan Primers for T2R16	ThermoFisher Scientific	Hs00249955_s1
TaqMan Primers for T2R19	ThermoFisher Scientific	Hs05000933_s1
TaqMan Primers for T2R20	ThermoFisher Scientific	Hs00604340_s1
TaqMan Primers for T2R30	ThermoFisher Scientific	Hs03054740_sH
TaqMan Primers for T2R31	ThermoFisher Scientific	Hs00604313_sH
TaqMan Primers for T2R38	ThermoFisher Scientific	Hs00604294_s1
TaqMan Primers for T2R39	ThermoFisher Scientific	Hs00603443_s1
TaqMan Primers for T2R40	ThermoFisher Scientific	Hs00602589_s1
TaqMan Primers for T2R41	ThermoFisher Scientific	Hs00603461_s1
TaqMan Primers for T2R42	ThermoFisher Scientific	Hs00704057_s1
TaqMan Primers for T2R43	ThermoFisher Scientific	Hs00853105_sH

TaqMan Primers for T2R45	ThermoFisher Scientific	Hs00820227_s1
TaqMan Primers for T2R46	ThermoFisher Scientific	Hs00853124_s1
TaqMan Primers for T2R50	ThermoFisher Scientific	Hs00604351_s1
TaqMan Primers for T2R60	ThermoFisher Scientific	Hs00603474_s1
TaqMan Primers for UBC	ThermoFisher Scientific	Hs01871556_s1
Recombinant DNA		
nls-R-GECO	Addgene	32462
pECFP-DEVD-Venus	Addgene	24537
pECFP-DEVG-Venus	Addgene	34538
pcDNA3-FlipGFP-T2A-mCherry	Addgene	124434
pCX-SpiCee-NLS	Addgene	140900
pCX-SpiCee-NES	Addgene	140901
pRS hTAS2R4 shRNA	Origene	TR301233B
pRS hTAS2R14 shRNA	Origene	TR301238A
Software		
MetaFluor	Molecular Devices	N/A
MetaMorph	Molecular Devices	N/A
QuantStudio 5	Applied Biosystems, Inc	N/A
Prism v8	GraphPad Software	N/A
ImageJ/FIJI	Open Source [2]	N/A
cBio Cancer Genomics Portal	cbioportal.org [3, 4]	N/A

Supplementary Table S2 Clinical data for HNSCC patients

Subject	Age	Sex	Race	Primary Site	Subsite	p16 status	Pathologic stage
1	69	F	white	oral cavity	oral tongue	n/a	T1N0M0
2	62	M	white	oral cavity	oral tongue	n/a	T2N0M0
3	72	M	white	oral cavity	retromolar trigone	n/a	T2N0M0
4	65	M	white	oropharynx	base of tongue	p16+	T2N3M0
5	59	M	white	oropharynx	base of tongue	p16+	T3N0M0
6	52	F	white	oropharynx	base of tongue	p16+	T4N2M0 (recurrent)
7	65	M	white	oropharynx	tonsil	p16+	T2N0M0
8	57	M	white	oropharynx	tonsil	p16+	T2N2M0
9	66	M	white	oropharynx	parapharyngeal space[a]	p16-	T1N1M0 (recurrent)
10	64	M	white	oropharynx	tonsil	n/a	T2N2bM0

Clinical data for head and neck squamous cell carcinoma (HNSCC) patients included in quantitative PCR (qPCR) taste receptor expression analysis (Figure 2). Includes 3 patients with oral cavity cancer and 7 patients with oropharyngeal cancer. Pathologic staging based on the American Joint Committee on Cancer (AJCC) 8th edition TNM staging [5]. [a]Primary tumor was located in the tonsil with recurrence/metastasis to a parapharyngeal space lymph node.

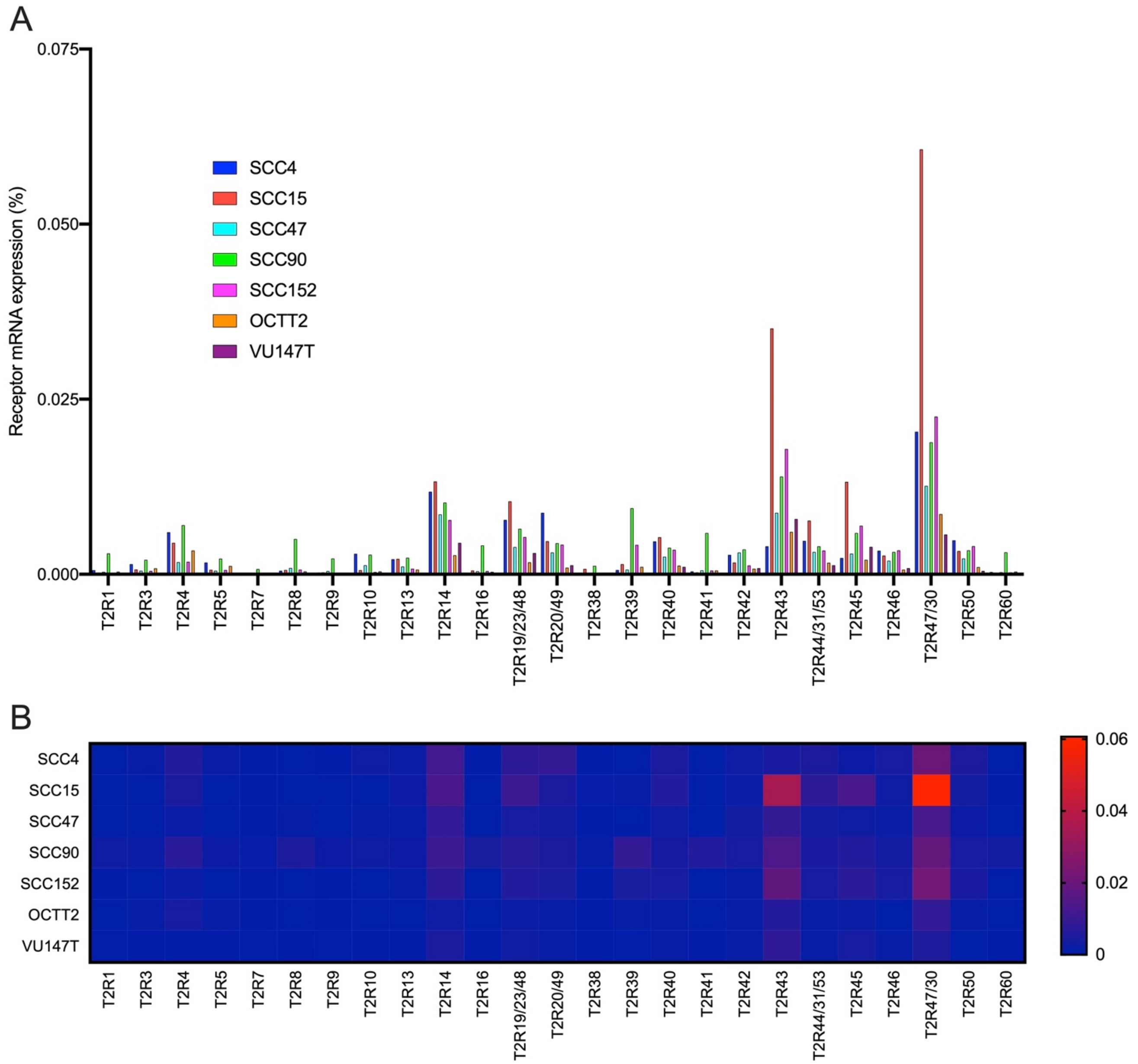

Supplementary Fig. S1 Variable mRNA expression of bitter (T2R) taste receptor genes in head and neck squamous cell carcinoma (HNSCC) cell lines. (A) Plot and (B) heatmap of quantitative PCR expression analysis of T2R transcripts in HNSCC cell lines SCC4, SCC15, SCC47, SCC90, SCC152, OCTT2, and VU147T shown as relative expression normalized to ubiquitin C (UBC) housekeeping gene.

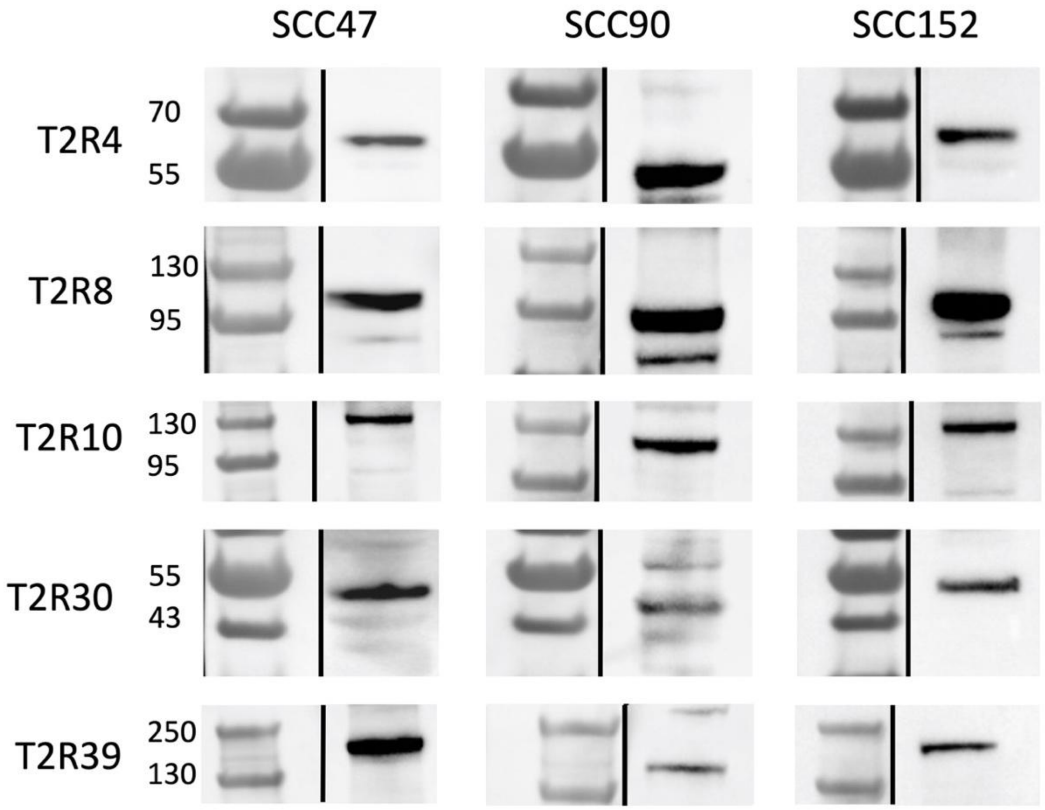

Supplementary Fig. S2 Confirmation of endogenous T2R protein expression by Western blot. Endogenous T2Rs were detected by Western as previously described (preprint [6]). Cells were lysed and run on a NuPage 4-12% Bis-Tris gel, transferred to nitrocellulose, then blocked in 5% milk in 50 mM Tris, 150 mM NaCl, and 0.025% Tween-20 (Tris-Tween) for 1 hour. Samples were not boiled as boiling promotes T2R GPCR aggregation (our experience), thus MW likely represents dimer or multimer forms of the protein. Primary antibody (1:1000) incubation in Tris-Tween with 5% BSA was 1.5 hours. Goat anti-rabbit or anti-mouse IgG-horseradish peroxidase secondary antibodies (1:5000) was for 1 hour. Blots were visualized with Clarity ECL on an imager with Image Lab Software (BioRad). These T2Rs were chosen because the antibodies were validated in our lab using knockout and/or heterologous expression studies [7-9, 6]. Not all T2Rs detected by qPCR could be examined via Western.

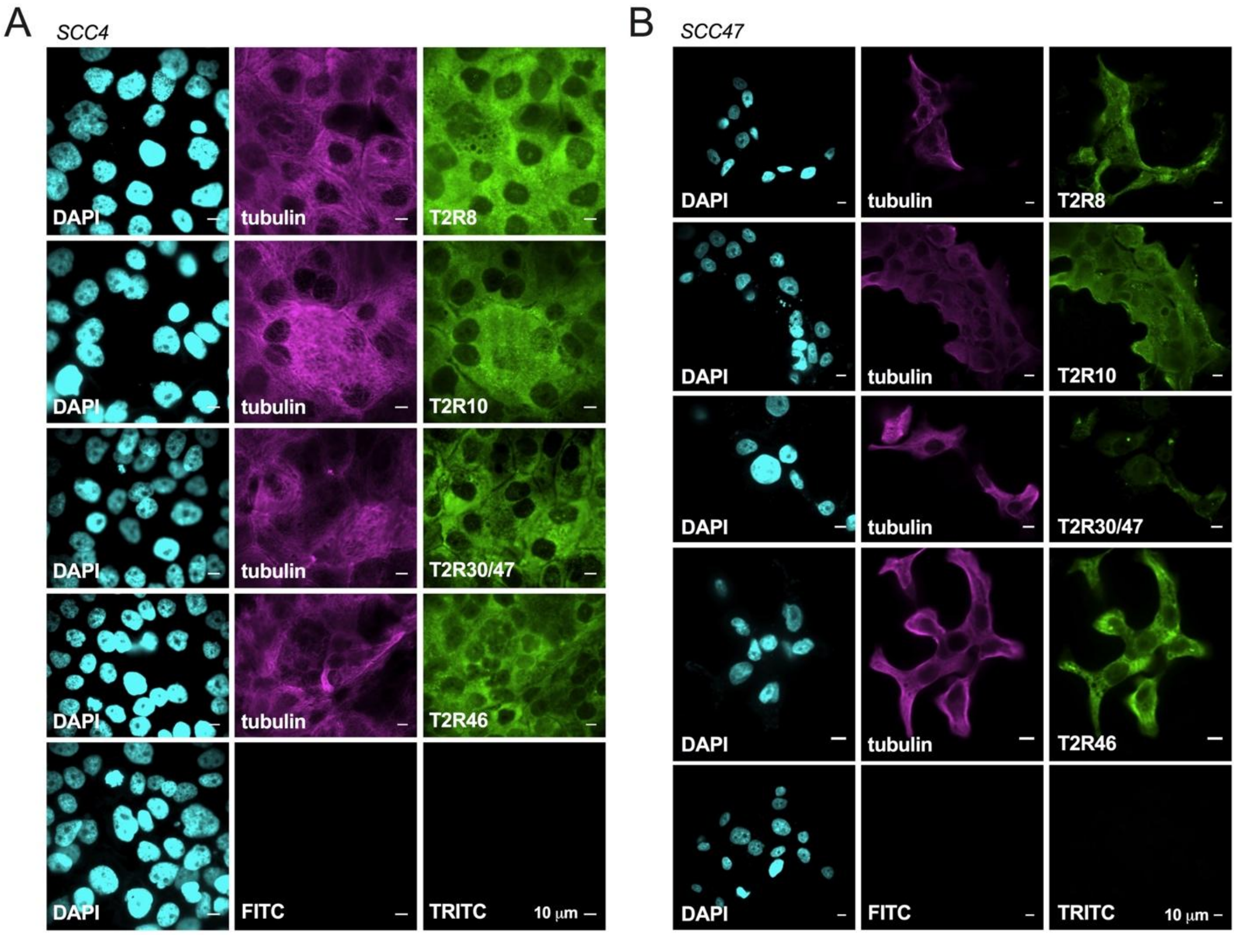

Supplementary Fig. S3 T2Rs are expressed in head and neck squamous cell carcinoma (HNSCC) cell lines. Fixed cultures of HNSCC cell lines SCC4 (A) and SCC47 (B) stained with antibodies targeting endogenous proteins demonstrate that T2Rs 8, 10, 30/47, and 46 localize to the plasma membrane. For all images, 1 representative image from 3 experiments were shown. Each antibody was compared to secondary only control at the same microscope settings.

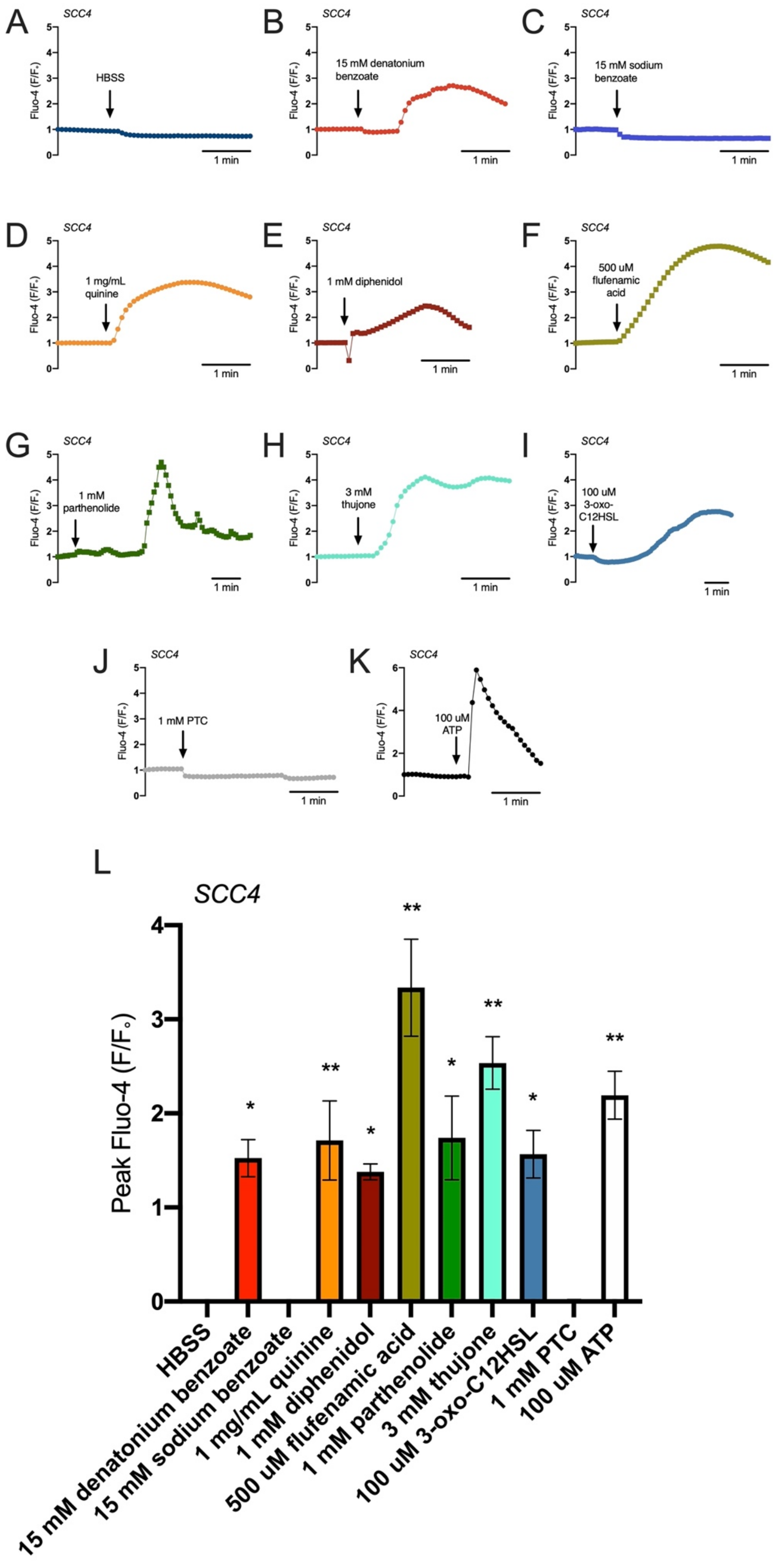
A
SCC4
HBSS
Fluo-4 (F/F₀)
1 min
B
SCC4
15 mM denatonium benzoate
C
SCC4
15 mM sodium benzoate
D
SCC4
1 mg/mL quinine
E
SCC4
1 mM diphenidol
F
SCC4
500 uM flufenamic acid
G
SCC4
1 mM parthenolide
H
SCC4
3 mM thujone
I
SCC4
100 uM 3-oxo-C12HSL
J
SCC4
1 mM PTC
K
SCC4
100 uM ATP
L
SCC4
Peak Fluo-4 (F/F₀)
HBSS
15 mM denatonium benzoate
15 mM sodium benzoate
1 mg/mL quinine
1 mM diphenidol
500 uM flufenamic acid
1 mM parthenolide
3 mM thujone
100 uM 3-oxo-C12HSL
1 mM PTC
100 uM ATP

Supplementary Fig. S4 Bitter (T2R) agonists activate calcium responses in head and neck squamous cell carcinoma (HNSCC) cell line. A-K HNSCC cell line SCC4 loaded with Ca^{2+} binding dye, Fluo-4, was stimulated with T2R agonists and Ca^{2+} was measured over time. Representative traces are shown after stimulation with Hank's Balanced Salt Solution (HBSS; control) (A), denatonium benzoate (B), sodium benzoate (C), quinine (D), diphenidol (E), flufenamic acid (F), parthenolide (G), thujone (H), N-3-oxo-dodecanoyl-L-homoserine lactone (3-oxo-C12HSL) (I), phenylthiocarbamide (PTC) (J), and purinergic receptor agonist adenosine triphosphate (ATP) (K). **L** Peak Fluo-4 F/F_o was quantified and compared to HBSS (mean ± SEM; 3-7 experiments using separate cultures). Significance by 1-way ANOVA with Bonferroni post-test. Peak Fluo-4 F/F_o was quantified after stimulation with Hank's Balanced Salt Solution (HBSS), denatonium benzoate, sodium benzoate, quinine, diphenidol, flufenamic acid, parthenolide, thujone, N-3-oxo-dodecanoyl-L-homoserine lactone (3-oxo-C12HSL), phenylthiocarbamide (PTC), and purinergic receptor agonist adenosine triphosphate (ATP) (mean ± SEM; 3-7 experiments using separate cultures). Significance by 1-way ANOVA with Bonferroni post-test comparing HBSS to each agonist. *p<0.05; **p<0.01.

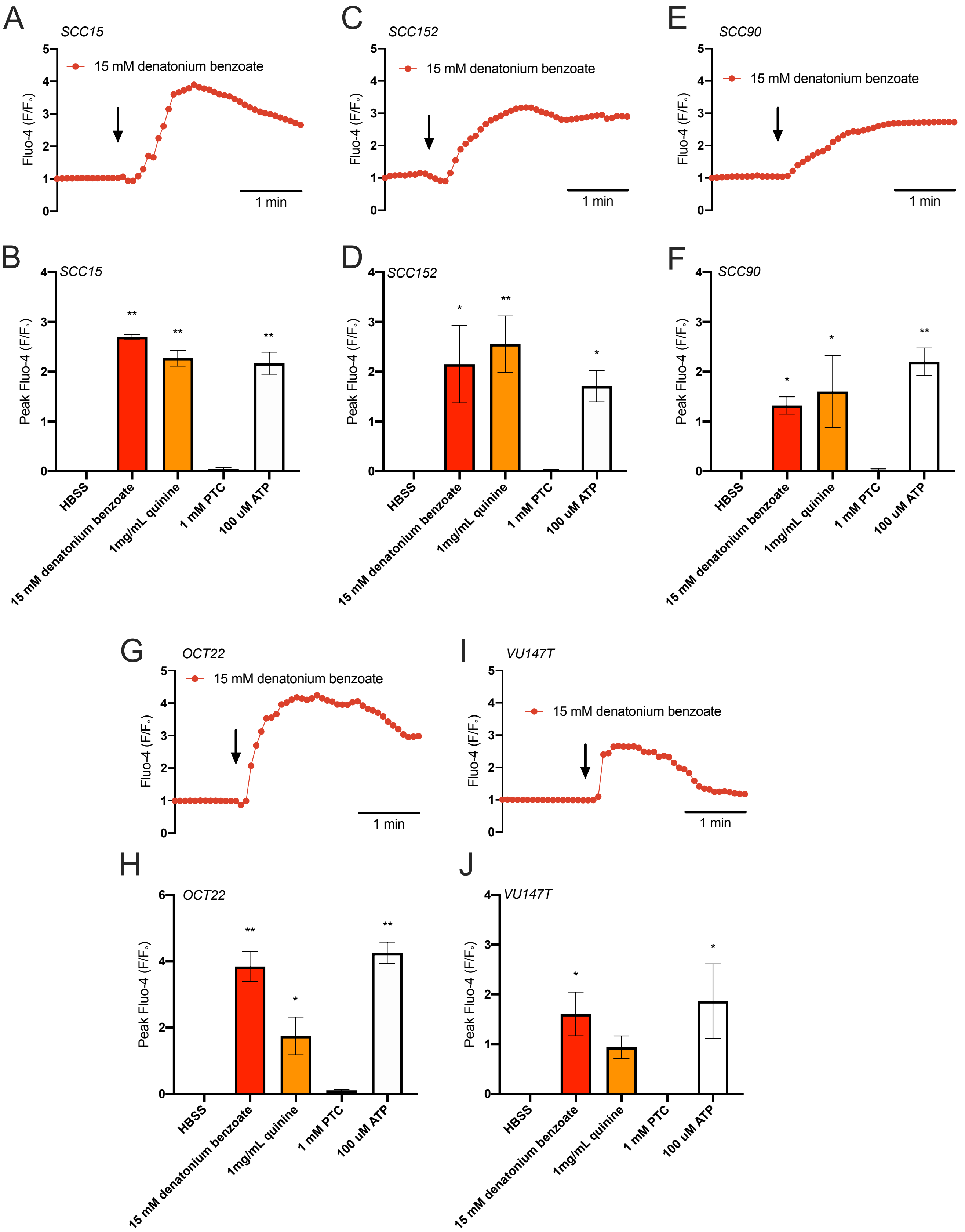
A
SCC15
15 mM denatonium benzoate
Fluo-4 (F/F₀)
1 min
B
SCC15
Peak Fluo-4 (F/F₀)
HBSS
15 mM denatonium benzoate
1mg/mL quinine
1 mM PTC
100 uM ATP
C
SCC152
15 mM denatonium benzoate
Fluo-4 (F/F₀)
1 min
D
SCC152
Peak Fluo-4 (F/F₀)
E
SCC90
15 mM denatonium benzoate
Fluo-4 (F/F₀)
1 min
F
SCC90
Peak Fluo-4 (F/F₀)
G
OCT22
15 mM denatonium benzoate
Fluo-4 (F/F₀)
1 min
H
OCT22
Peak Fluo-4 (F/F₀)
I
VU147T
15 mM denatonium benzoate
Fluo-4 (F/F₀)
1 min
J
VU147T
Peak Fluo-4 (F/F₀)

Supplementary Fig. S5 Bitter (T2R) agonists activate calcium responses in head and neck squamous cell carcinoma (HNSCC). HNSCC cell lines loaded with Fluo-4 were stimulated with denatonium benzoate (agonist for T2R4, 8, 10, 13, 39, 43, 46, and 47), quinine (agonist for T2R4, 7, 10, 14, 39, 40, 43, 44, and 46), phenylthiocarbamide (PTC; agonist for T2R38), and adenosine triphosphate (ATP; agonist for purinergic receptors) and calcium responses were measured over time. Representative traces after stimulation with denatonium benzoate from single cultures of SCC15 (A), SCC152 (C), SCC90 (E), OCT22 (G), and VU147T (I). Peak Fluo-4 F/F_o was quantified for each cell line (SCC15 (B), SCC152 (D), SCC90 (F), OCT22 (H), and VU147T (J)) after stimulation with Hank's Balanced Salt Solution (HBSS), denatonium benzoate, quinine, PTC, and ATP (mean ± SEM; 3-7 experiments using separate cultures for each cell line). Significance by 1-way ANOVA with Bonferroni post-test comparing HBSS to each agonist. **p*<0.05; ***p*<0.01.

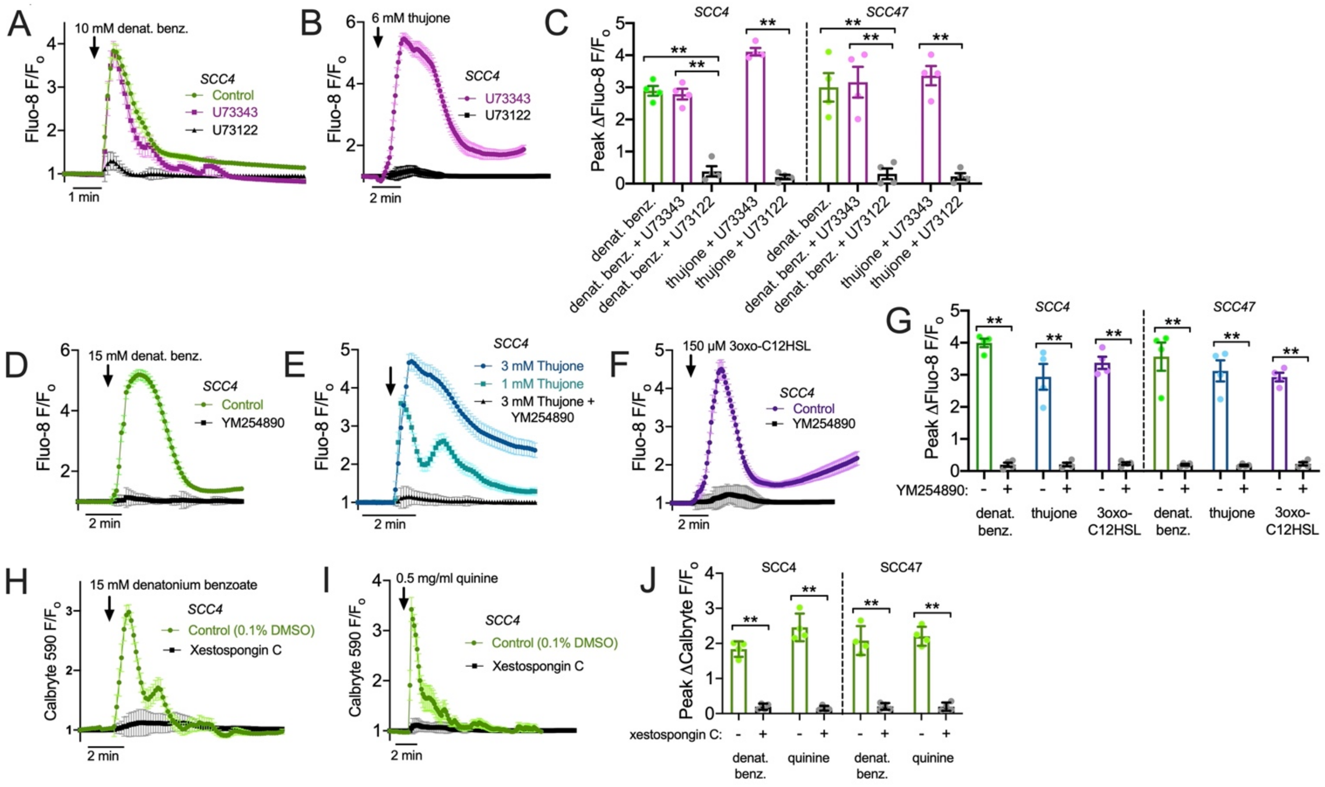

Supplementary Fig. S6 Pharmacology of the Ca^{2+}_i response. A-C SCC4 and SCC47 cells were loaded with Fluo-8 for 45 min and imaged as described in the text. Cells were loaded in the presence of 0.1% DMSO (vehicle control), 10 µM phospholipase C inhibitor U73122, or 10 µM inactive analogue U73343. Cells were stimulated with denatonium benzoate or thujone as indicated in the continued presence or absence of inhibitor. Representative traces are shown from SCC4 for denatonium (A) and thujone (B). Bar graph (C) shows inhibition of Ca^{2+}_i responses by U73122 but not U73343. **D-G** SCC4 and SCC47 cells were loaded with Fluo-8 for 45 min and imaged as described. After loading, cells were incubated with 0.1% DMSO (vehicle control) or 10 µM YM254890, a heterotrimeric G protein inhibitor. Cells were then stimulated with denatonium, thujone, or 3-oxo-C12HSL. Representative traces from SCC4 shown in D, E, and F, respectively. Bar graph (G) shows inhibition of Ca^{2+}_i with all agonists in the presence of YM254890. **H-J** SCC4 and SCC47 cells were loaded with calcium indicator Calbryte 590 for 45 min and imaged as described in the text. Cells were pre-incubated for 10 min with 0.1% DMSO (vehicle control) or 10 µM xestospongin C, and IP_3R inhibitor. (H) and (I) show representative traces from SCC4 cells stimulated with denatonium or quinine, respectively. Bar graph (J) shows reduced Ca^{2+}_i response with xestospongin C. All traces shown are representative experiments showing mean ± SEM of 20-40 cells from a single field of view. Data points in bar graphs are independent experiments (n = 3-6). Significance determined by one-way ANOVA with Bonferroni posttest; ***p*<0.01.

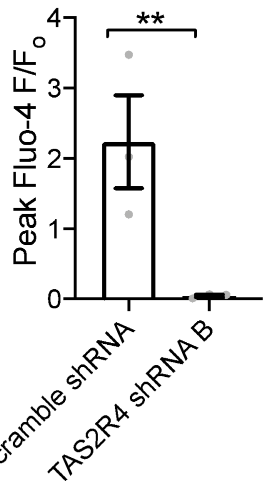

Supplementary Fig. S7 Inhibition of denatonium-induced fluo-4 Ca^{2+} responses with knockdown of T2R4. Stable cells SCC90 cells were made by transfection of pRS vector containing shRNA for *TAS2R4* or scramble shRNA followed by puromycin selection. qPCR quantified level of *TAS2R4* mRNA knockdown was 97%. Peaks from independent Fluo-4 experiments were quantified. Bar graph shows mean ± SEM. Significance by Student's t test; ***p*<0.01. Denatonium response was almost completely eliminated with knockdown of *TAS2R4*.

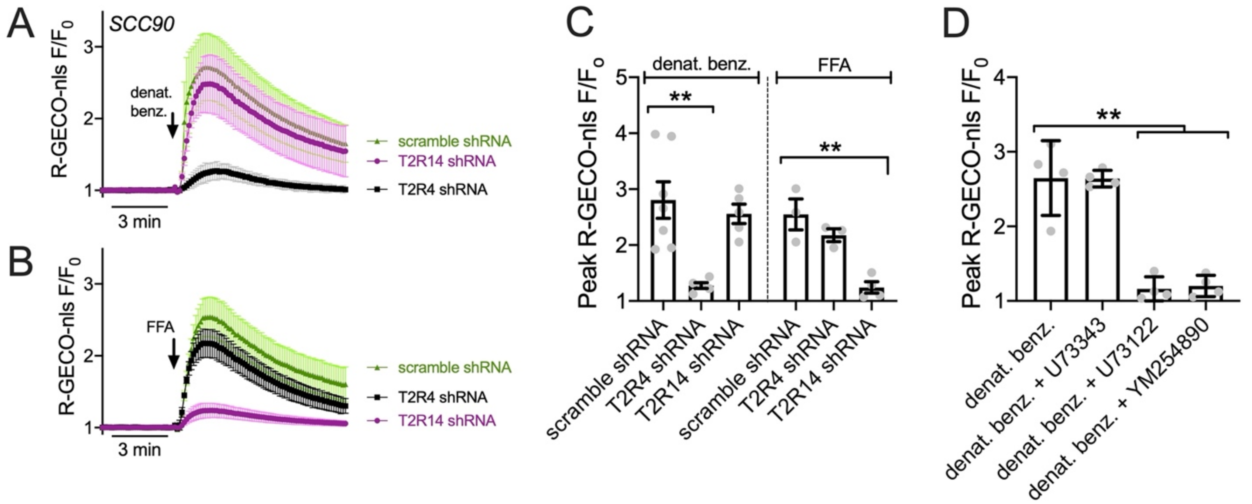

Supplementary Fig. S8 Inhibition of denatonium-induced or flufenamic acid (FFA)-induced R-GECO-nls Ca^{2+}_{nuc} responses with *TAS2R4* or *TAS2R14* shRNA, respectively. SCC90 cells were co-transfected with R-GECO-nls and shRNA plasmids as indicated. Cells were imaged after 72 hours and stimulated with 10 mM denatonium benzoate or 500 µM FFA. **A-B** Average traces from n = 5-7 experiments per condition. **C** Quantificiation of peaks from individual experiments showing reduction of denatonium response with *TAS2R4* shRNA and reduction of FFA response with *TAS2R14* shRNAs. Significance by one way ANOVA with Bonferonni posttest; **p<0.01. **D** Bar graph showing R-GECO-nls experiments with denatonium benzoate ± PLC inhibitor U73122 (or inactive control U73343) or G protein inhibitor YM254890, used as described in Supplementary Fig. S6. Note reduction of denatonium-induced R-GECO-nls responses with U73122 and YM254890.

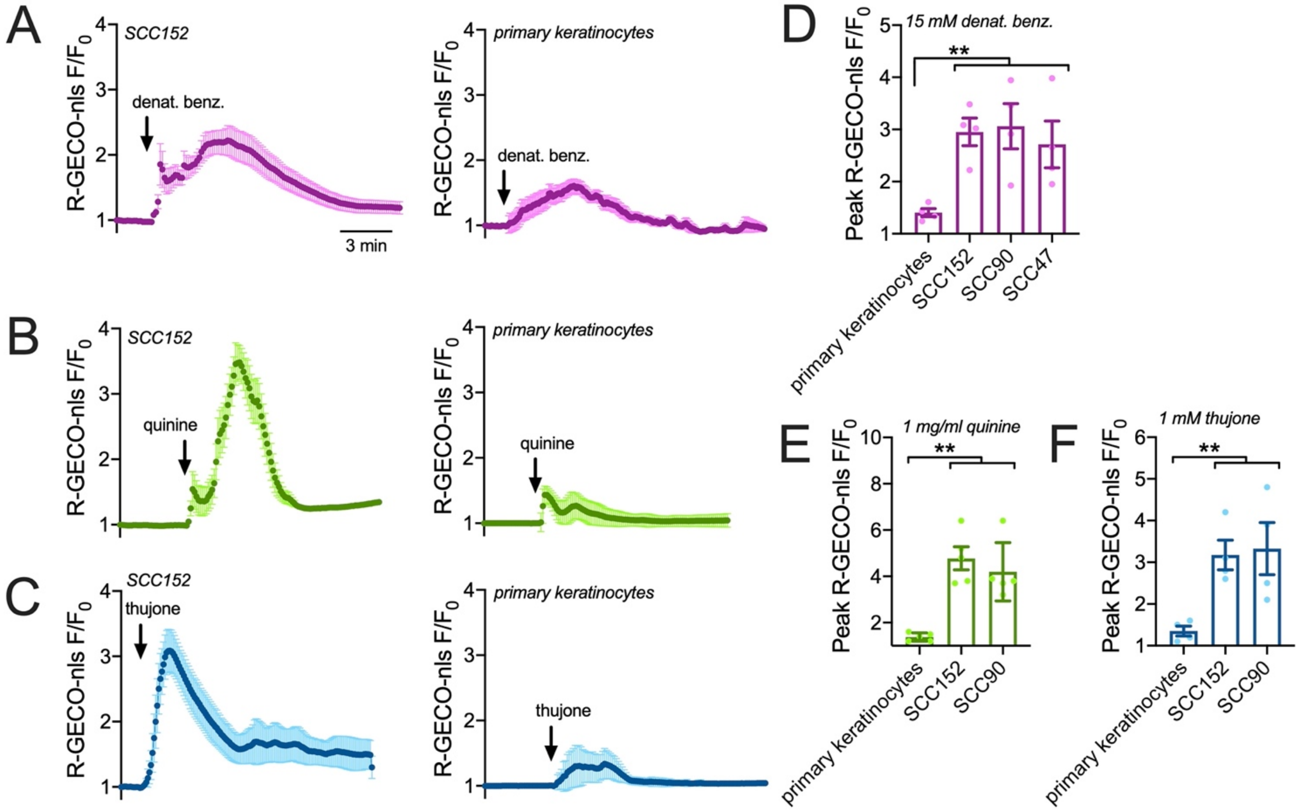

Supplementary Fig. S9 Reduced Ca^{2+}_{nuc} responses in primary oral keratinocytes compared with HNSCC cells. **A-B** Representative R-GECO-nls responses to denatonium benzoate (A), quinine (B), and thujone (C) in SCC152 cells and primary keratinocytes. Experiments performed as described in the main text (Figure 4F-G). Cells were imaged 48 hours post transfection. **D-F** shows quantification of independent experiments (n = 4, from different donors for primary keratinocytes). Significance by one-way ANOVA with Bonferonni posttest. Note larger Ca^{2+}_{nuc} responses in HNSCC cells vs primary keratinocytes with all three T2R agonists.

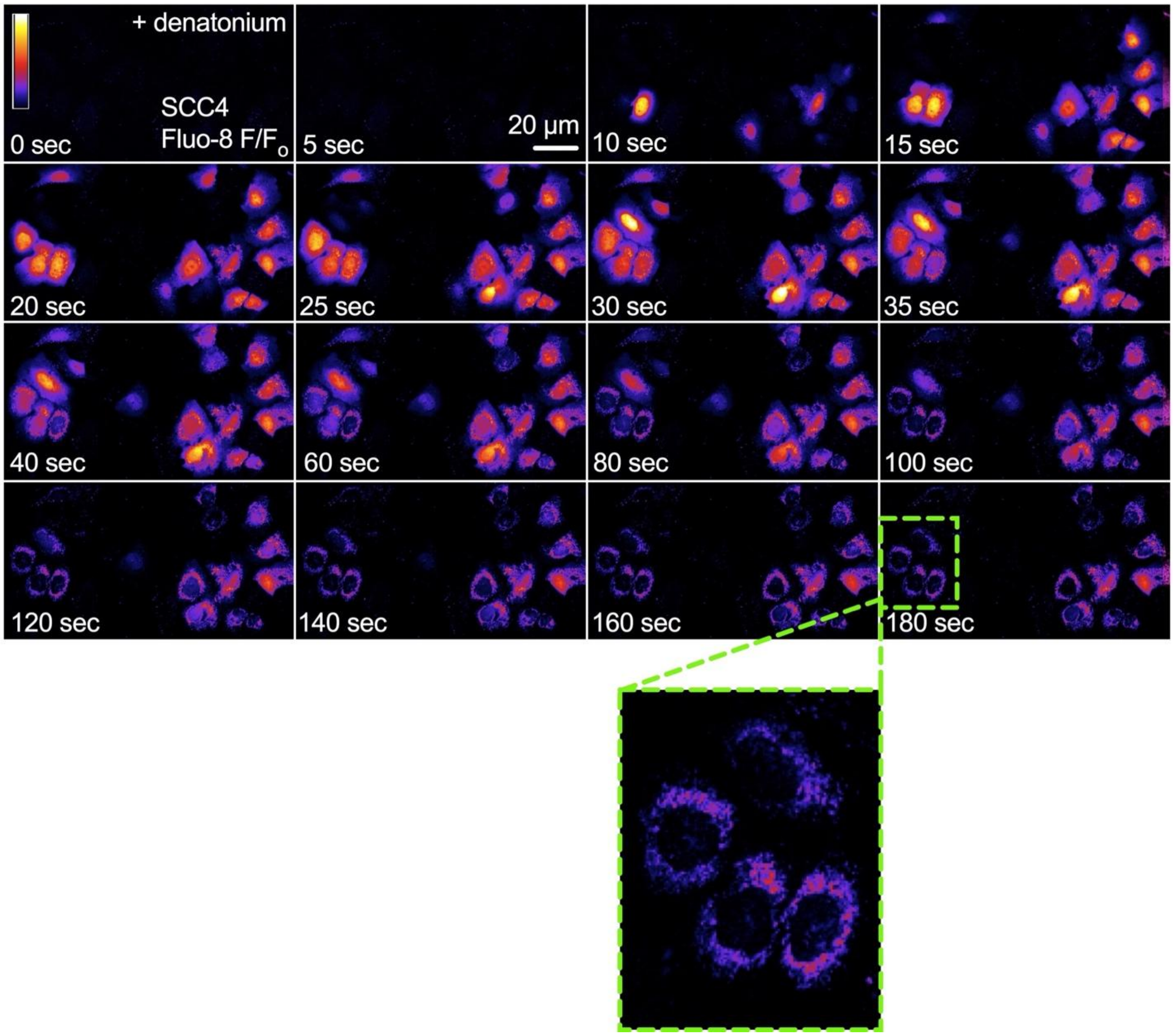

Supplementary Fig. S10 Nuclear Ca^{2+} responses appeared to propagate to mitochondria in Fluo-8-loaded cells. Representative F/F$_o$ images of a time course of denatonium stimulation in SCC4 cells loaded with Fluo-8. The initial nuclear Ca^{2+} increase was followed by a lower-level but more sustained increase in Ca^{2+} in a perinuclear pattern reminiscent of mitochondria. Green outlined box at bottom shows enlargement of outlined region from 180 sec time point. These observations suggested that bitter agonist-activated nuclear Ca^{2+} signals might influence mitochondrial function.

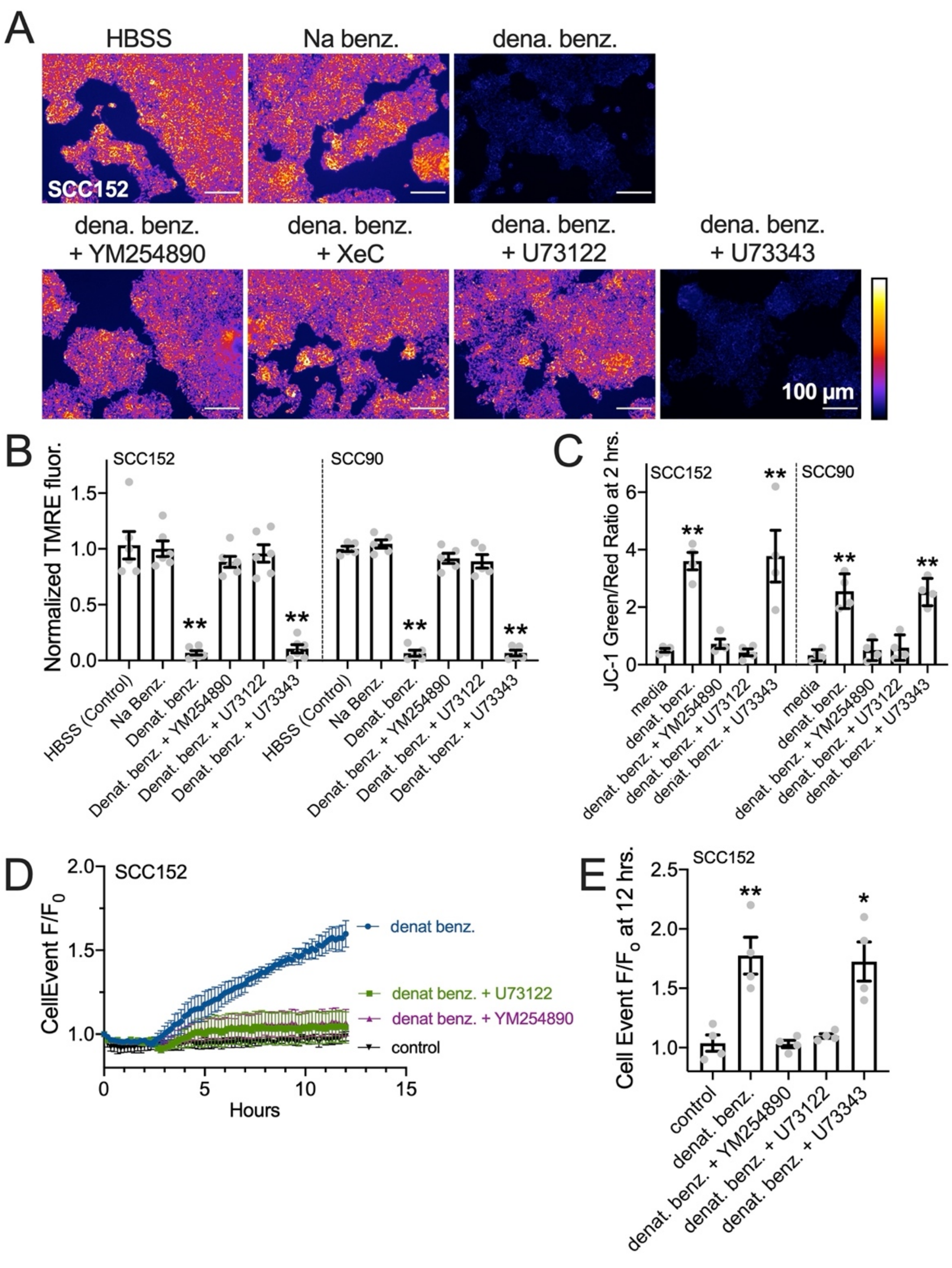

Supplementary Fig. S11 Inhibition of denatonium-induced TMRE, JC-1, and CellEvent changes by GPCR inhibitors. A-B TMRE mitochondrial membrane potential assays were performed as described in the main text (Fig 5E-H) using 10 mM denatonium benzoate or sodium benzoate and GPCR inhibitors as described in Supplementary Figure S6. Representative TMRE images are shown in A and quantification of fluorescence intensity from individual independent experiments (n = 6) are shown in B. Significance in B by one way ANOVA with Dunnett's posttest comparing all values to

control (HBSS only). ***p*<0.01. Note inhibition of denatonium-reduced TMRE fluorescence with YM254890 and U73122. **C** JC-1 mitochondrial membrane potential assay was performed similarly to the main text but as an end point assay. Green/red fluorescence ratio was quantified after 3 hours. Note reduction of denatonium-induced depolarization (increased green fluorescence) with YM584890 and U73122. Bar graph shows mean ± SEM from n = 4 independent experiments. Significance by one way ANOVA with Dunnett's posttest comparing all values to control (HBSS only); ***p*<0.01. **D-E** CellEvent caspase activation assays were performed as described in the main text. D shows representative trace and E shows bar graph of mean ± SEM from independent experiments. Significance by one way ANOVA with Dunnett's posttest comparing all values to control (HBSS only); ***p*<0.01. Note reduction of denatonium-induced caspase activation with U73122 and YM254890.

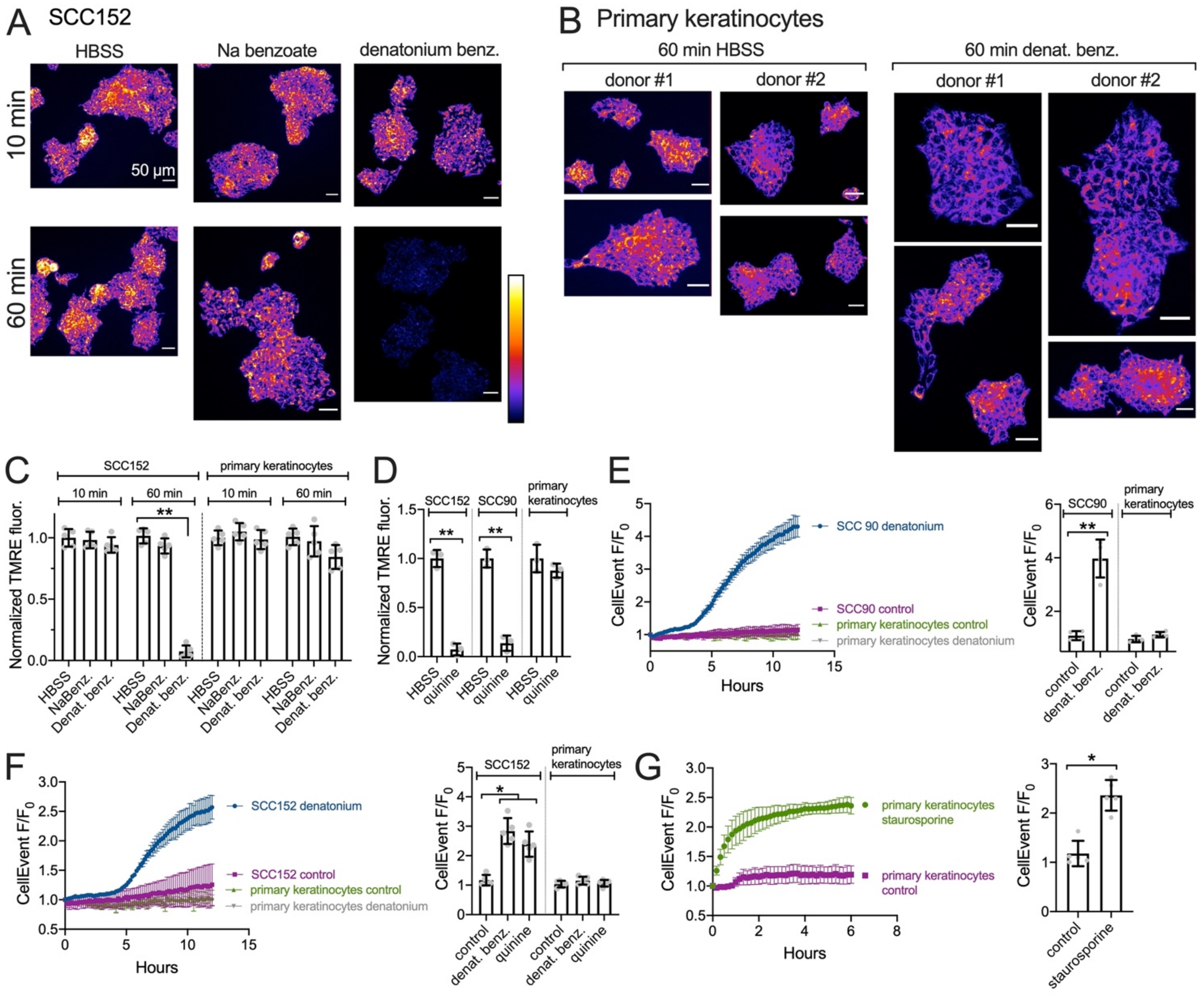

Supplementary Fig. S12 Lack of effect of T2R stimulation on TMRE, JC-1, and CellEvent fluorescence in primary keratinocytes. **A-B** Representative images of TMRE fluorescence after stimulation with 10 mM denatonium benzoate in SCC152 cells (A) and primary keratinocytes (B). **C** Bar graph shows quantification from n = 5 independent experiments as in A-B (using cells from multiple donors in the case of keratinocytes). Significance by Bonferonni posttest comparing values to respective control (HBSS at that time point); $^{**}p<0.01$. Note loss of TMRE fluorescence with denatonium after 60 min in SCC152 but not primary cells. **D** Experiments were performed and quantified similarly to A-C but with 0.5 mg/ml quinine. Note loss of TMRE fluorescence with quinine after 60 min in SCC152 and SCC90 but not primary cells. Significance by Bonferonni posttest comparing values to respective control (HBSS at that time point); $^{**}p<0.01$. **E-F** CellEvent caspase

activation assays were performed simultaneously in primary keratinocytes and SCC90 (E) or SCC152 (F) HNSCC cells. Traces are from representative experiments and bar graph show mean ± SEM from n = 5 independent experiments. Significance by one ways ANOVA with Bonferroni posttest comparing all values to respective control (HBSS only). Note caspase activation in HNSCC but not primary cells. **G** As a positive control for apoptosis in primary keratinocytes, we used PKC inhibitor staurosporine. Representative experiment and bar graph of independent experiments (n = 5) shown. Significance by Student's t test; **$p<0.01$

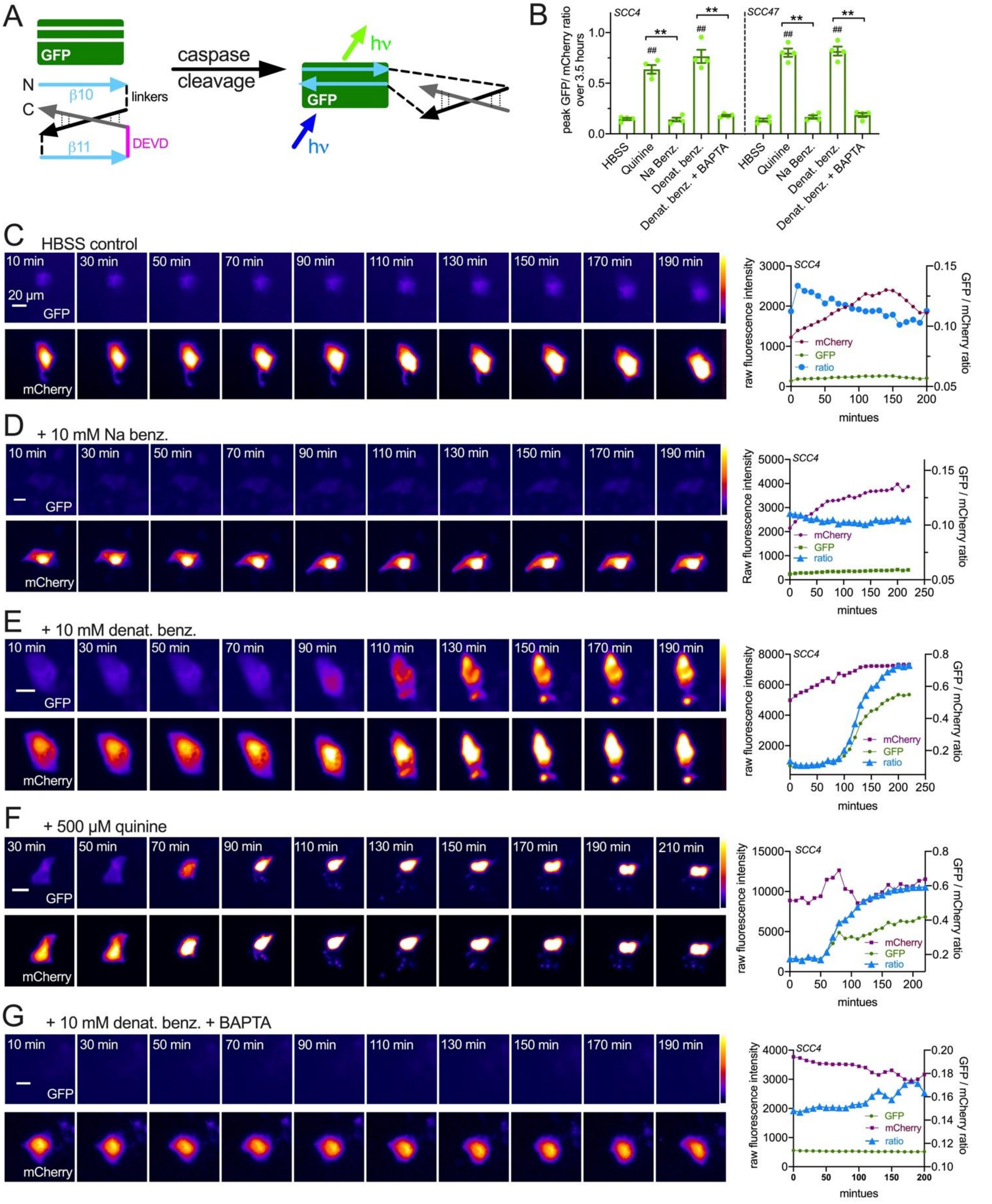
A
GFP
N
β10
linkers
C
β11
DEVD
caspase
cleavage
hν
GFP
hν
B
SCC4
SCC47
peak GFP/ mCherry ratio over 3.5 hours
HBSS
Quinine
Na Benz.
Denat. benz.
Denat. benz. + BAPTA
C HBSS control
20 µm
GFP
mCherry
raw fluorescence intensity
GFP / mCherry ratio
mintues
D + 10 mM Na benz.
E + 10 mM denat. benz.
F + 500 µM quinine
G + 10 mM denat. benz. + BAPTA
ratio

Supplementary Fig. S13 Confirmation of caspase activation by Flip-GFP and dependence of denatonium-induced caspase activation on Ca^{2+} signaling. **A** Diagram of the Flip-GFP assay [10]. GFP ®10 and ®11 strands are anti-parallel within the GFP ® barrel. The Flip construct contains parallel ®10 and ®11 joined by a linker. Cleavage of the DEVD sequence in the construct permits flipping of ®11, allowing ®10 and ®11 to fit into the GFP barrel and complete the GFP. Thus, caspase activity will increase GFP fluorescence. Soluble mCherry is expressed as a transfection control. **B** Cells were imaged with a 10x objective and GFP and mCherry fluorescence was measured in single cells over 3-4 hours (representative experiments below). Background-subtracted fluorescence ratios are shown after 3.5 hours (210 min) stimulation as indicated. BAPTA-loaded cells were pre-incubated with 10 µM of global calcium chelator BAPTA-AM for 1 hour. Cells for all other conditions were similarly incubated in the absence of BAPTA). Each data point represents one cells from an independent experiment (n=3-5). Bar graph is mean ± SEM. Significance by one way ANOVA with Bonferonni posttest; ## $p<0.01$ vs HBSS control; ** $p<0.01$ vs bracketed bars. **C-G** Representative intensity pseudocolored images of GFP and mCherry fluorescence (left) as well as traces showing fluorescence changes in SCC4 cells during incubation with HBSS (control; C), sodium benzoate (D), denatonium benzoate (E), quinine (F), or denatonium + BAPTA (G). Background subtraction and quantification of cell fluorescence was carried out using ImageJ.

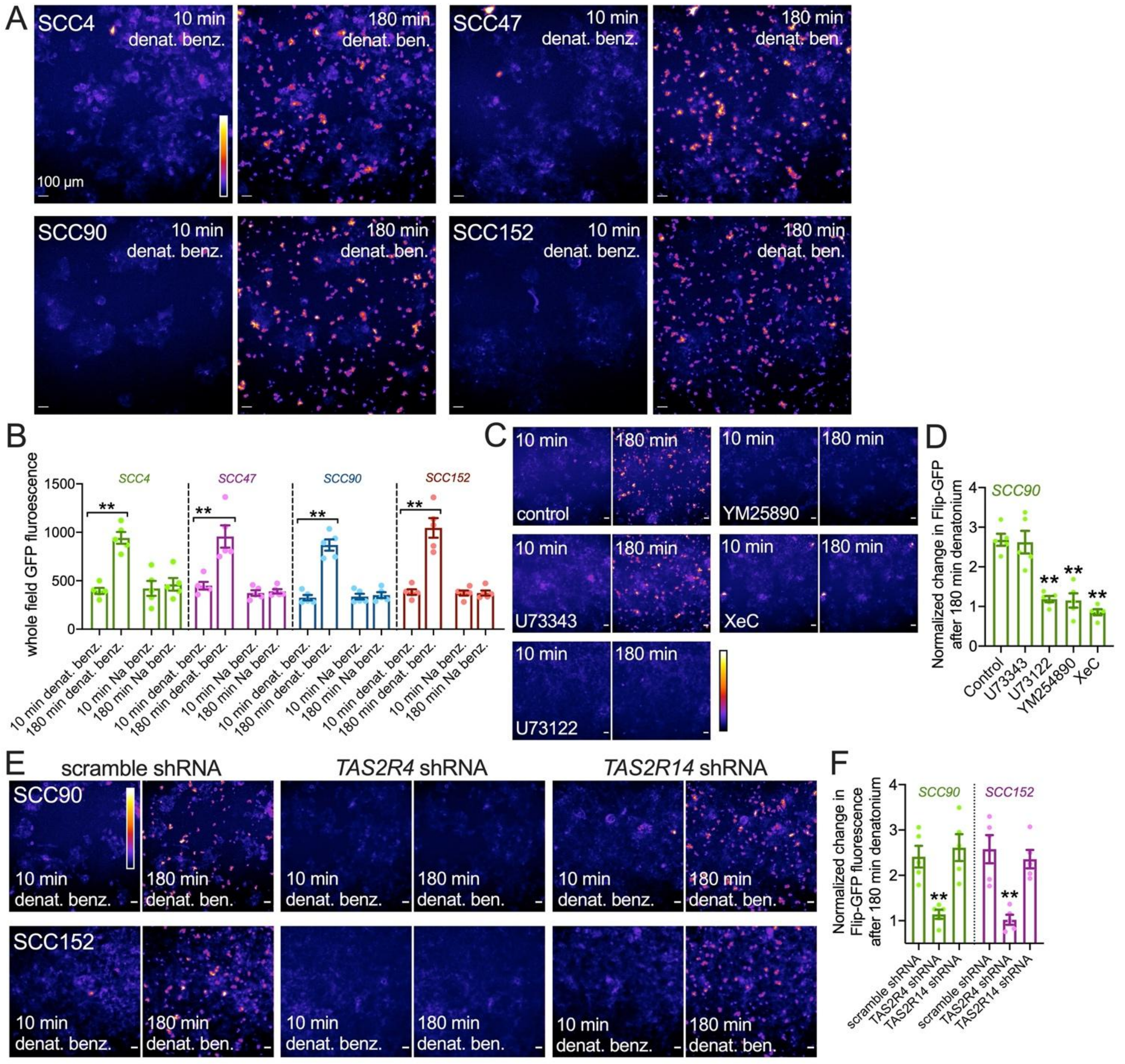

Supplementary Fig. S14 Flip-GFP measurement of caspase activation in response to denatonium benzoate but not sodium benzoate in SCC4, SCC47, SCC90, and SCC152. Cells were imaged using 4x objective and GFP fluorescence was estimated from whole field fluorescence intensity. **A** Representative intensity pseudocolored images of GFP fluorescence at 10 min and 180 min after denatonium benzoate stimulation in four different cell lines as indicated. **B** Bar graph of quantified data from experiments in (A) where cells were stimulated with 15 mM denatonium benzoate or sodium benzoate. Each data point represents one cells from an independent experiment (n=3-5).

Bar graph is mean ± SEM. Significance by one way ANOVA with Bonferonni posttest; ** p<0.01 vs bracketed bars. Background subtraction and quantification of cell fluorescence was carried out using ImageJ. **C-D** Experiments (n = 5) as in A-B with denatonium benzoate ± U73343, U73122, YM25890, or XeC, as described in Supplementary Fig. S9. U73122, YM25890 and XeC reduced caspase activation. Significance in bar graph by one-way ANOVA with Dunnett's posttest comparing all values to control (HBSS only); ***p*<0.01. **E-F** Experiments (n = 5) as above with denatonium benzoate ± co-transfection with scramble, *TAS2R4*, or *TAS2R14* shRNA. Note inhibition of denatonium-induced caspase activation with *TAS2R4* but not *TAS2R14* shRNA. Significance in bar graph by one-way ANOVA with Dunnett's posttest comparing all values to control (HBSS only); ***p*<0.01.

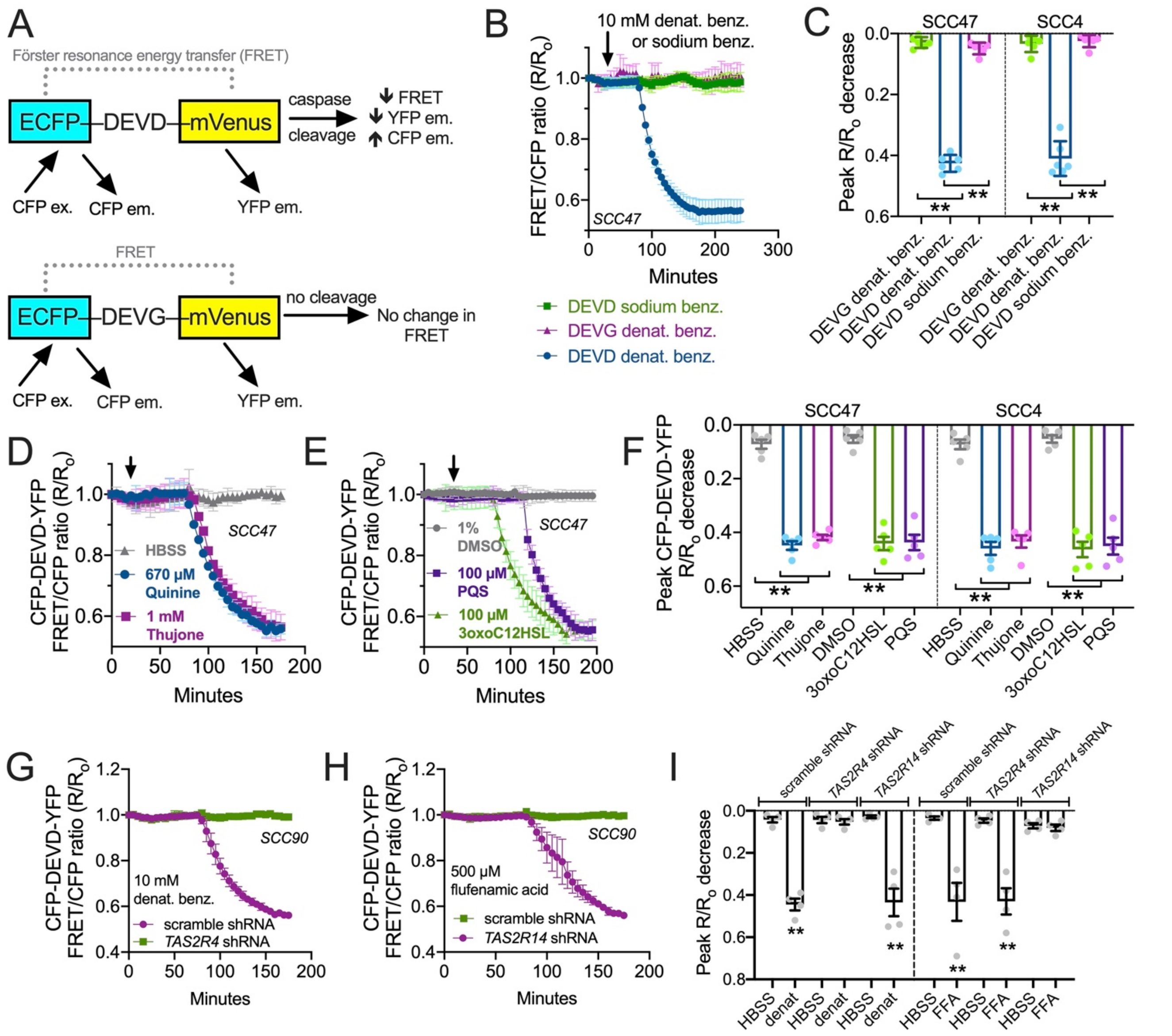

Supplementary Fig. S15 Confirmation of bitter agonist-induced caspase activation by ratiometric caspase biosensor. **A** Schematic of the biosensor (top) showing enhanced CFP (ECFP) connected to YFP variant mVenus by a DEVD linker. Caspase cleavage allows the ECFP and mVenus to diffuse apart and reduces FRET signal (YFP emission with CFP excitation). A control biosensor (bottom) was used with the DEVD replaced with a non-cleavable DEVG. **B** Representative traces (mean ± SEM of 5-9 transfected cells) from SCC47 cells showing decreased FRET after ~60 min denatonium benzoate stimulation with DEVD biosensor but not uncleavable control DEVG biosensor. Sodium benzoate had no effect. **C** Bar graph showing peak FRET decrease in SCC47 and

SCC4 cells from independent experiments as in (B). **D-E** Representative traces of DEVD biosensor FRET ratio in SCC47 cells stimulated with quinine (D), thujone (D), Pseudomonas quinolone signal (PQS; (E)), 3-oxo-C12HSL (E), or 1% DMSO (vehicle control for 3-oxo-C12HSL and PQS; (E)). **F** Bar graph showing peak FRET decrease in SCC47 and SCC4 cells from independent experiments as in (D-E). Data points in bar graphs are independent experiments (n = 3-6). Significance determined by one-way ANOVA with Bonferonni posttest; ***p*<0.01. Together, these data support activation of caspases by multiple bitter agonists, including bacterial 3-oxo-C12HSL and PQS. **G-I** Similar experiments were performed with denatonium benzoate (G) or flufenamic acid (H) with co-transfection of *TAS2R4* or *TAS2R14* shRNA. Note that *TAS2R4* shRNA reduced denatonium caspase activation and *TAS2R14* shRNA reduced flufenamic acid caspase activation. I shows quantification of individual experiments (n = 4). Significance by one way ANOVA with Bonferroni posttest comparing HBSS vs denatonium or FFA for each condition; ***p*<0.01.

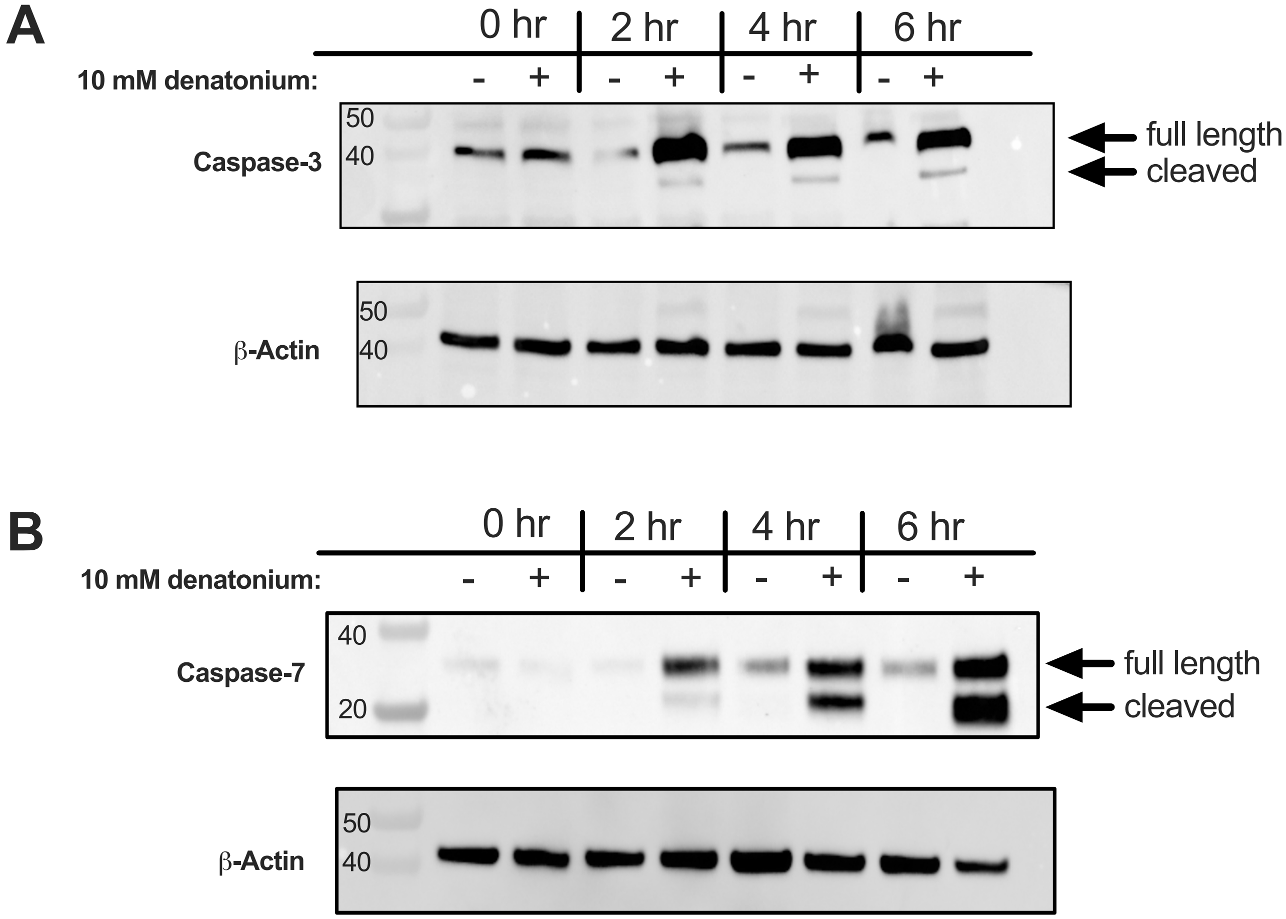

Supplementary Fig. S16 Confirmation of denatonium-induced caspase activation by Western for caspase 3 and 7 cleavage in SCC47 cells. Caspase 3 and 7 were detected using antibodies recognizing both full length and cleaved (activated) protein. After stimulation for the indicated times in the presence or absence of denatonium benzoate (in DMEM + 10% FBS), cells were lysed and run on a NuPage 4-12% Bis-Tris gel, transferred to nitrocellulose, then blocked in 5% milk in 50 mM tris, 150 mM NaCL, and 0.05% Tween-20 (Tris-Tween) for 1 hour. Primary antibody (1:1000) incubation in Tris-Tween with 5% BSA was 1 hour. Incubation with oat anti-rabbit or anti-mouse IgG-horseradish peroxidase secondary antibodies (1:1000) was 1 hour. Blots were visualized with either Clarity or ClarityMax ECL on an imager with Image Lab Software (BioRad). Gels shown are representative of 3 independent experiments using cells at different passage number on different days.

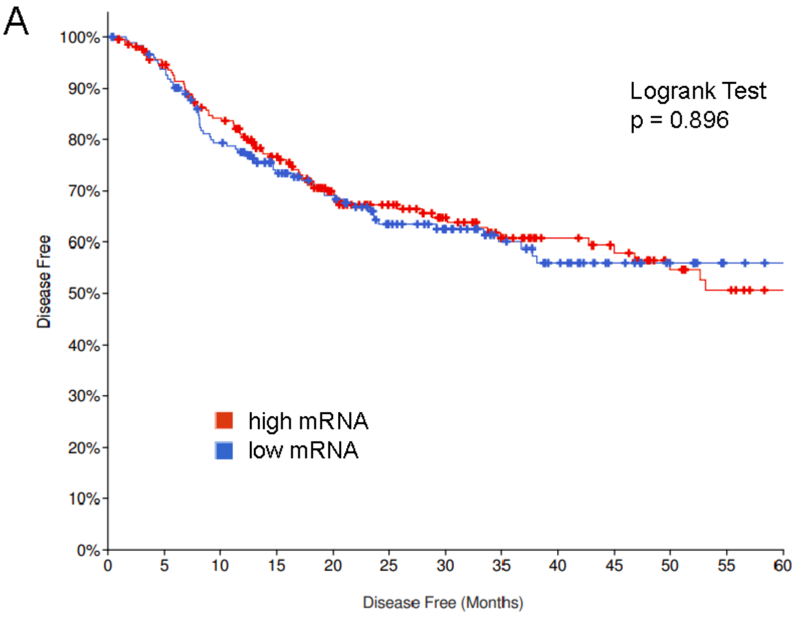

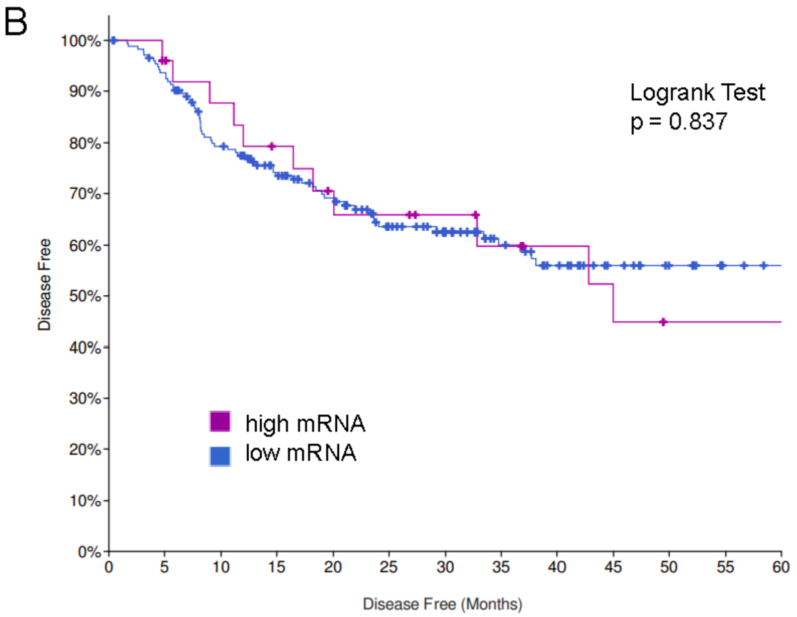

Supplementary Fig. S17 Disease-free survival for bitter taste receptor (*TAS2R*) expression alterations in head and neck squamous cell carcinoma (HNSCC). A 5-year disease-free survival analysis comparing HNSCC cases with increased TAS2R expression (p = 0.896 by logrank test) and **B** increased TAS2R4 expression (p = 0.837 by logrank test).

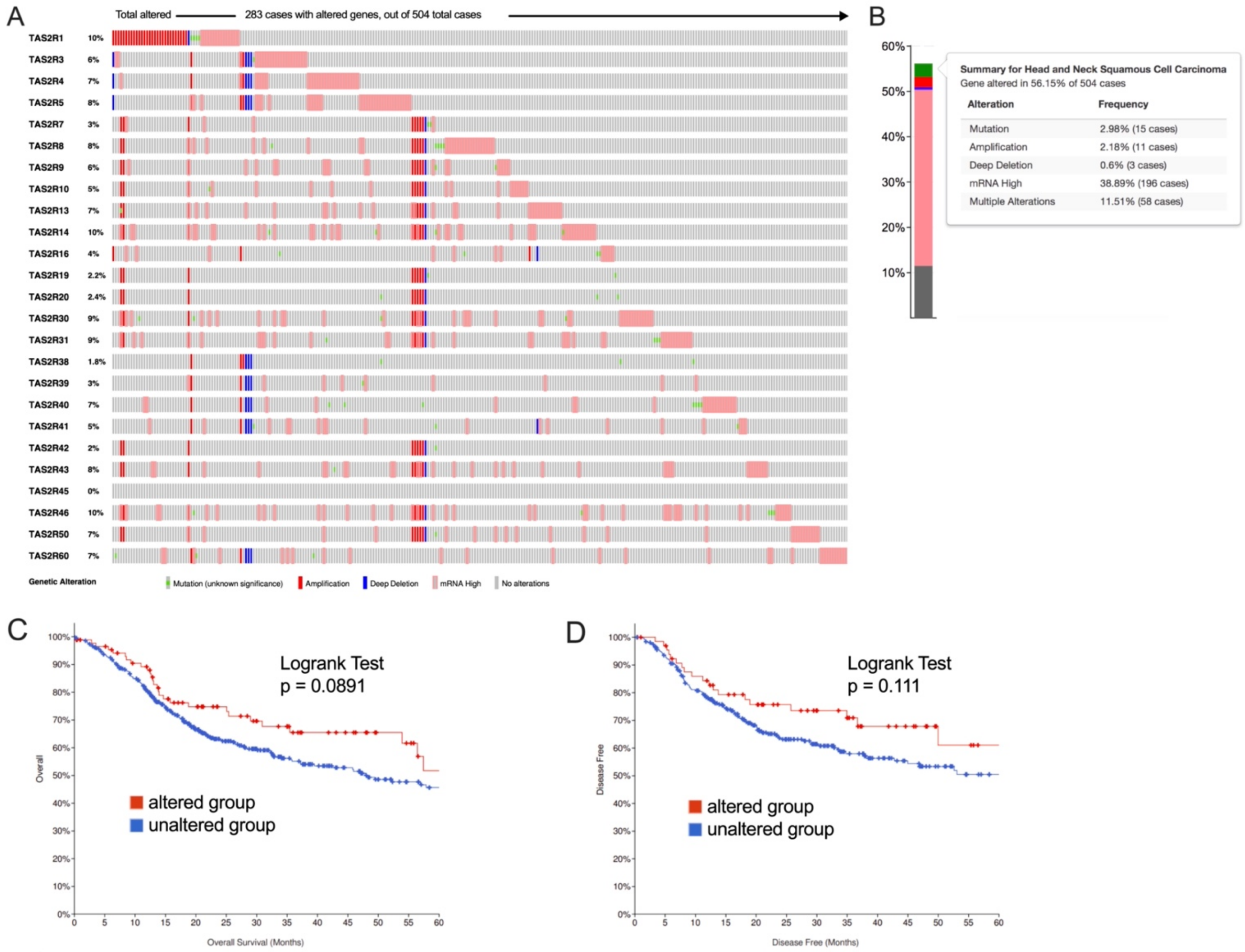

Supplementary Fig. S18 Bitter taste receptor (*TAS2R*) genomic and expression alterations are prevalent in head and neck squamous cell carcinoma (HNSCC). *TAS2R* genetic and expression alterations were analyzed for 504 cases of HNSCC using The Cancer Genome Atlas (TCGA) [3, 4]. A total of 283 out of 504 cases (56.15%) included some *TAS2R* genetic or expression alteration. **A** OncoPrint of *TAS2R* genomic and expression alterations in HNSCC. Genes are listed in rows with percentage of altered cases; individual cases are listed in columns. **B** Bar graph demonstrating alteration types and frequencies in HNSCC. Multiple alterations were present in 11.51% of cases. **C** 5-year overall survival analysis of *TAS2R* genetic alterations (not including expression changes) in HNSCC (p = 0.0891 by logrank test). **D** 5-year disease-free survival analysis of *TAS2R* genetic alterations (not including expression changes) in HNSCC (p = 0.111 by logrank test).

Supplementary References

1. Basu D, Nguyen TT, Montone KT, Zhang G, Wang LP, Diehl JA, Rustgi AK, Lee JT, Weinstein GS & Herlyn M (2010) Evidence for mesenchymal-like sub-populations within squamous cell carcinomas possessing chemoresistance and phenotypic plasticity. Oncogene 29, 4170-4182, doi: 10.1038/onc.2010.170.

2. Schindelin J, Arganda-Carreras I, Frise E, Kaynig V, Longair M, Pietzsch T, Preibisch S, Rueden C, Saalfeld S, Schmid B, Tinevez JY, White DJ, Hartenstein V, Eliceiri K, Tomancak P & Cardona A (2012) Fiji: an open-source platform for biological-image analysis. Nat Methods 9, 676-682, doi: 10.1038/nmeth.2019.

3. Cerami E, Gao J, Dogrusoz U, Gross BE, Sumer SO, Aksoy BA, Jacobsen A, Byrne CJ, Heuer ML, Larsson E, Antipin Y, Reva B, Goldberg AP, Sander C & Schultz N (2012) The cBio cancer genomics portal: an open platform for exploring multidimensional cancer genomics data. Cancer discovery 2, 401-404, doi: 10.1158/2159-8290.cd-12-0095.

4. Gao J, Aksoy BA, Dogrusoz U, Dresdner G, Gross B, Sumer SO, Sun Y, Jacobsen A, Sinha R, Larsson E, Cerami E, Sander C & Schultz N (2013) Integrative analysis of complex cancer genomics and clinical profiles using the cBioPortal. Sci Signal 6, pl1, doi: 10.1126/scisignal.2004088.

5. Amin MB, Greene FL, Edge SB, Compton CC, Gershenwald JE, Brookland RK, Meyer L, Gress DM, Byrd DR & Winchester DP (2017) The Eighth Edition AJCC Cancer Staging Manual: Continuing to build a bridge from a population-based to a more "personalized" approach to cancer staging. CA Cancer J Clin 67, 93-99, doi: 10.3322/caac.21388.

6. McMahon DB, Kuek LE, Johnson ME, Johnson PO, Horn RLJ, Carey RM, Adappa ND, Palmer JN & Lee RJ (2021) The bitter end: T2R bitter receptor agonists elevate nuclear calcium and induce apoptosis in non-ciliated airway epithelial cells. bioRxiv, 2021.2005.2016.444376, doi: 10.1101/2021.05.16.444376.

7. Freund JR, Mansfield CJ, Doghramji LJ, Adappa ND, Palmer JN, Kennedy DW, Reed DR, Jiang P & Lee RJ (2018) Activation of airway epithelial bitter taste receptors by Pseudomonas aeruginosa quinolones modulates calcium, cyclic-AMP, and nitric oxide signaling. J Biol Chem 293, 9824-9840, doi: 10.1074/jbc.RA117.001005.

8. Hariri BM, McMahon DB, Chen B, Freund JR, Mansfield CJ, Doghramji LJ, Adappa ND, Palmer JN, Kennedy DW, Reed DR, Jiang P & Lee RJ (2017) Flavones modulate respiratory epithelial innate immunity: anti-inflammatory effects and activation of the T2R14 receptor. J Biol Chem 292, 8484-8497, doi: 10.1074/jbc.M116.771949.

9. Lee RJ, Hariri BM, McMahon DB, Chen B, Doghramji L, Adappa ND, Palmer JN, Kennedy DW, Jiang P, Margolskee RF & Cohen NA (2017) Bacterial d-amino acids suppress sinonasal innate immunity through sweet taste receptors in solitary chemosensory cells. Sci Signal 10, doi: 10.1126/scisignal.aam7703.

10. Zhang Q, Schepis A, Huang H, Yang J, Ma W, Torra J, Zhang SQ, Yang L, Wu H, Nonell S, Dong Z, Kornberg TB, Coughlin SR & Shu X (2019) Designing a Green Fluorogenic Protease Reporter by Flipping a Beta Strand of GFP for Imaging Apoptosis in Animals. J Am Chem Soc 141, 4526-4530, doi: 10.1021/jacs.8b13042.
